# Dietary, lifestyle, and environmental factors associated with ulcerative colitis: a systematic review and meta-analysis of observational studies

**DOI:** 10.3389/fnut.2026.1885679

**Published:** 2026-07-24

**Authors:** Yahui Wang, Tianru Xie, Ziwen Liao, Sen He, Yumeng Zhao, Yi Li, Xiangying Kong, Pingliang Sun

**Affiliations:** 1 Guangxi University of Chinese Medicine, Nanning, Guangxi, China; 2 Department of Colorectal and Anal Surgery, The First Affiliated Hospital of Guangxi University of Chinese Medicine, Nanning, Guangxi, China

**Keywords:** dietary factors, environmental exposures, inflammatory bowel disease, lifestyle factors, meta-analysis, systematic review, ulcerative colitis

## Abstract

**Background and aims:**

Ulcerative colitis (UC) is increasing worldwide, and dietary, lifestyle, and environmental exposures may contribute to disease susceptibility. This systematic review and meta-analysis evaluated the associations between these exposures and the risk of incident UC.

**Methods:**

PubMed, Embase, and the Cochrane Library were searched for observational studies examining dietary, lifestyle, medication-related, surgical, early-life, sociodemographic, and other environmental exposures in relation to UC risk through November 12, 2025. Random-effects models were used to pool effect estimates. Study quality was assessed using the Newcastle–Ottawa Scale, and the certainty of evidence was evaluated using GRADE.

**Results:**

Thirty observational studies involving 148,468 patients with UC were included, covering 17 factors and 27 comparisons. Daily egg consumption, family history of inflammatory bowel disease, the highest reported category of antibiotic exposure, ever smoking, and former smoking were associated with increased UC risk. Pet exposure, coffee consumption, appendectomy, higher educational attainment, light alcohol consumption, and fruit intake were associated with reduced UC risk. Current smoking was associated with a lower risk of UC in case–control studies, whereas the association was not statistically significant in cohort studies. Adjusted estimates suggested that breastfeeding might be associated with a lower risk of UC, although the evidence was limited. No clear associations were observed for body mass index, oral contraceptive use, tonsillectomy, previous gastrointestinal infection, place of residence, any antibiotic use, tea consumption, or heavy alcohol consumption.

**Conclusion:**

Dietary, lifestyle, and broader environmental factors showed different directions of association with UC risk, although the overall certainty of evidence was low. These findings support further prospective and mechanistic studies to clarify causal pathways and identify potentially modifiable exposures relevant to UC prevention.

**Systematic review registration:**

PROSPERO, CRD420261343412.

## 1 Introduction

Ulcerative colitis (UC) is a chronic, idiopathic inflammatory disease of the colon clinically characterized by recurrent diarrhea, rectal bleeding, and abdominal pain, and is associated with substantial long-term morbidity and systemic health consequences (1). The prevalence of UC is highest in early-industrialized regions, including Europe, North America, and Oceania, but remains comparatively lower in newly industrialized or developing regions such as Asia, Africa, and Latin America (2). Epidemiological data further demonstrate marked geographic disparities in disease burden, with Europe reporting the highest prevalence of UC (198.6 per 100,000; 95% confidence interval [CI], 181.6–215.6) and Asia showing a substantially lower prevalence (22.4 per 100,000; 95% CI, 9.5–32.2) (3). Such regional variation suggests that the development of UC is shaped not only by host susceptibility but also by dietary patterns, lifestyle behaviors, and broader environmental exposures. Given the steadily increasing global burden of UC, a rigorous synthesis of dietary, lifestyle, and environmental factors associated with disease onset is important for informing primary prevention, risk stratification, and patient counseling.

UC is a multifactorial disease whose pathogenesis is thought to involve a complex interplay among genetic susceptibility, dietary and lifestyle exposures, broader environmental influences, gut microbial dysbiosis, and aberrant immune responses. In recent years, increasing attention has been directed toward dietary, lifestyle-related, medication-related, early-life, and sociodemographic determinants of inflammatory bowel disease (IBD). However, UC and Crohn’s disease (CD), although both classified within IBD, differ substantially in disease distribution, pathological behavior, and clinical course. UC is generally limited to the rectum and colon and manifests as continuous mucosal inflammation extending proximally from the rectum, whereas CD may involve any segment of the gastrointestinal tract and is characterized by discontinuous, transmural inflammation, with a higher risk of structuring, penetrating disease, and fistula formation (4, 5). Therefore, evidence syntheses that combine UC and CD under a broad IBD outcome may obscure UC-specific associations and reduce the clinical interpretability of findings. Unlike previous reviews that often evaluated IBD as a combined endpoint or focused on selected exposures, this systematic review and meta-analysis specifically investigated dietary, lifestyle, and environmental factors associated with the risk of UC. Crude and adjusted effect estimates were analyzed separately, with adjusted estimates prioritized when available. We further assessed the certainty of evidence using the GRADE framework, aiming to provide a UC-specific and methodologically rigorous evidence synthesis relevant to disease etiology, prevention, and clinical counseling.

## 2 Methods

This study was registered in the International Prospective Register of Systematic Reviews (PROSPERO; CRD420261343412) and was designed and conducted in accordance with the Preferred Reporting Items for Systematic Reviews and Meta-Analyses (PRISMA) statement. As this study did not involve the recruitment of human participants, approval from a local ethics committee was not required.

### 2.1 Search strategy

A systematic literature search was conducted in PubMed, Embase, and the Cochrane Library from inception to November 12, 2025. The search strategy combined the terms “ulcerative colitis,” “risk factor,” and “case–control study,” together with the corresponding Medical Subject Headings (MeSH) terms where applicable. No lower date limit was imposed at the search stage to ensure comprehensive retrieval of potentially relevant studies. However, to improve the contemporaneity and methodological relevance of the included evidence, and to reduce the potential impact of outdated methodologies and conclusions from earlier publications, only studies published between 2005 and 2025 were ultimately considered eligible. Conference abstracts, letters, editorials, and non-original articles were excluded. The full search strategies for each database are presented in Appendix 1 of the Supplementary material.

### 2.2 Study selection

Following removal of duplicate records, all retrieved studies were independently screened by two trained reviewers (Ziwen Liao and Tianru Xie) according to predefined inclusion and exclusion criteria, with each reviewer blinded to the other’s judgments throughout the selection process. Discrepancies were resolved through discussion with a senior investigator (Pingliang Sun) until consensus was achieved. The selection process comprised three stages: (1) title and abstract screening; (2) full-text assessment; and (3) a final eligibility check during data extraction. Studies for which the required data could not be obtained from the original reports or from the corresponding authors were excluded.

### 2.3 Eligibility criteria

Studies were considered eligible if they met the following criteria: (1) evaluated potentially relevant dietary, lifestyle, medication-related, surgical, early-life, sociodemographic, or other environmental exposures; (2) included patients with a clear diagnosis of UC based on internationally accepted diagnostic criteria; (3) were original observational studies, specifically case–control or cohort studies; and (4) reported sufficient effect estimate data on the association between these exposures and UC risk to permit quantitative synthesis.

Studies were excluded if they were (1) reviews, systematic reviews, meta-analyses, book chapters, or other non-original publications; (2) animal, preclinical, or mechanistic studies without data on the association between these exposures and UC risk; (3) studies focusing exclusively on CD or other non-UC diseases, or studies that did not report UC-specific data separately; or (4) duplicate publications or reports based on overlapping datasets.

### 2.4 Data extraction

Two authors (Yahui Wang and Yumeng Zhao) independently extracted the data, and disagreements were resolved by discussion or, if required, by consultation with a third investigator until consensus was achieved. Extracted information included the first author, publication year, study design, geographic region, study period, total sample size, numbers of events in the case and control groups, participants’ age, exposure factors, adjusted covariates, and effect estimates (ORs, RRs, or HRs with corresponding 95% CIs). When necessary, the corresponding authors were contacted for additional information. Study quality was assessed using the Newcastle–Ottawa Scale (NOS), and the certainty of evidence was evaluated according to the GRADE framework.

### 2.5 Statistical analysis

All statistical analyses were conducted using StataNow 19 MP. Random-effects models were applied to pool effect estimates for the associations between individual exposure factors and the risk of UC. Effect measures were analyzed separately, and ORs, RRs, and HRs were not combined in the same meta-analysis. Smoking comparisons were analyzed separately according to the original study-reported contrasts (ever vs. never, current vs. never, former vs. never, and current vs. non-current smoking) and were not combined across different reference groups. Between-study heterogeneity was quantified using the *I*^2^ statistic. For comparisons with substantial heterogeneity (*I*^2^ > 65%) and at least three included studies, leave-one-out sensitivity analyses were conducted to assess the influence of individual studies on the pooled estimate and heterogeneity. For comparisons including only two studies, leave-one-out sensitivity analysis was not performed because exclusion of either study would preclude pooled estimation. Where data permitted, analyses were stratified by exposure definition, effect estimate type, or study design. Meta-regression was not performed because the number of studies within individual comparisons was insufficient. Potential small-study effects were explored by visual inspection of funnel plots for meta-analyses including at least three studies. Egger’s test was not performed because no comparison included 10 or more studies, below which the test has limited statistical power. A two-sided *p* value of <0.05 was considered statistically significant. Details of leave-one-out sensitivity analyses are provided in Appendix 6 of the Supplementary material.

## 3 Results

### 3.1 Search results

**Figure 1:**
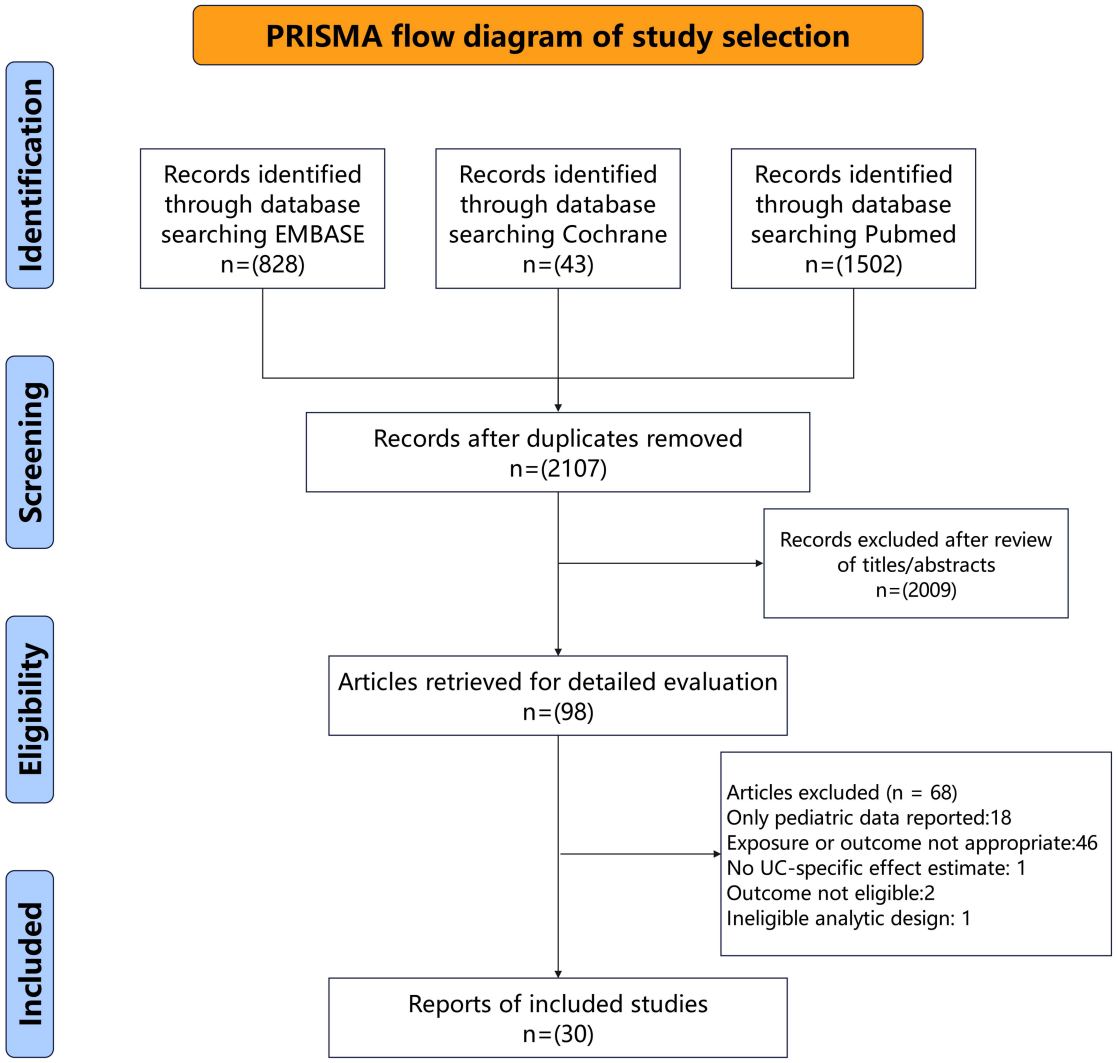
PRISMA flow diagram of the study selection process.

The database search identified 2,373 records from Embase, the Cochrane Library, and PubMed. After duplicate removal, 2,107 records remained for title and abstract screening, and 98 articles were subsequently reviewed in full text. Of these, 30 studies fulfilled the eligibility criteria and were included in the meta-analysis (Figure 1), representing 148,468 participants in total. The included studies spanned more than 40 years of clinical data collection, covering the period from 1980 to 2021. These studies were geographically diverse, involving 17 countries—the United Kingdom, Canada, Iran, France, China, Spain, New Zealand, Denmark, Australia, the United States, Sweden, India, South Korea, Brazil, the Netherlands, Japan, and Türkiye—as well as one multicountry European study. The study populations also covered a broad age spectrum: the mean or median age of patients with UC ranged from approximately 30.0 to 65.0 years, and several studies enrolled participants from around 18 years to over 90 years of age (Tables 1, 2).

**Table 1:** Methodological characteristics and quality assessment of included studies.

Study	Study design	Exposure factors assessed, *n*	Effect measures reported	Study quality (NOS)
GarciaRodriguez_2005 (24)	Case–control	3	aOR	High
Bernstein_2006 (25)	Case–control	5	Mixed (aOR + OR)	High
Firouzi_2006 (26)	Case–control	4	aOR	Moderate
deSaussure_2007 (27)	Case–control	2	OR	High
Jiang_2007 (28)	Case–control	5	aOR	High
Sicilia_2008 (29)	Case–control	2	OR	High
LopezSerrano_2010 (30)	Case–control	4	aOR	High
Gearry_2010 (31)	Case–control	5	aOR	High
Hansen_2011 (32)	Case–control	6	OR	High
Chan_2013 (33)	Cohort	2	aOR	High
Wang_2013 (34)	Case–control	4	OR	High
Ko_2015 (35)	Case–control	6	aOR	High
Niewiadomski_2016 (36)	Case–control	4	OR	Moderate
Niu_2016 (37)	Case–control	3	aOR	High
Aniwan_2018 (38)	Case–control	1	aOR	High
Axelrad_2018 (39)	Case–control	1	aOR	High
Amarapurkar_2018 (40)	Case–control	2	OR	Moderate
Park_2019 (9)	Cohort	1	aHR	High
Salgado_2020 (41)	Case–control	5	aOR	High
van_der_Sloot_2020 (42)	Case–control	9	aOR	High
Nguyen_2020 (43)	Case–control	1	aOR	High
Blackwell_2021 (44)	Case–control	1	aOR	High
Oh_2023 (45)	Case–control	1	aOR	High
Tanaka_2023 (6)	Case–control	2	aOR	High
Kondo_2023 (46)	Case–control	3	aOR	High
Sun_2023 (47)	Cohort	1	aHR	High
Alperen_2024 (7)	Case–control	8	aOR	High
Chu_2024 (48)	Case–control	1	aOR	High
Ho_2025 (49)	Cohort	1	aHR	High
Ahn_2025 (50)	Cohort	1	aHR	High

**Table 2:** Study population characteristics of included studies.

Study	Country/region	Study period (years)	Total participants, *N*	UC cases/events, *n*	Age characteristics of UC group (years)	Non-cases/controls, *n*	Age characteristics of control group (years)
GarciaRodriguez_2005	UK	1995–1997	10,222	222	20–84	1,000	20–84
Bernstein_2006	Canada	NR	650	217	18–50	433	18–50
Firouzi_2006	Iran	NR	764	382	40.0 ± 14.6	382	40.0 ± 14.6
deSaussure_2007	France	2003(June–Dec)	396	198	43.7 ± 14.3	198	43.9 ± 14.2
Jiang_2007	China	2004 (Jan–Dec)	354	177	42.9 ± 15.5	177	42.9 ± 15.1
Sicilia_2008	Spain	1992–1995	410	250	34.9 ± 15.4	250	35.5 ± 15.0
LopezSerrano_2010	Spain	during 2004	424	146	49.7 ± 16.6	278	Age-matched
Gearry_2010	New Zealand	2003–2005	1,253	653	≥20	600	≥20
Hansen_2011	Denmark	2003–2004	288	144	38.5 (13–95)	144	40.5 (11–86)
Chan_2013	Europe	1991–2004	885	177	52.8 (22.0–77.0)	708	52.7 (22.0–77.2)
Wang_2013	China	2007–2010	2,616	1,308	41.6 ± 12.3	1,308	41.4 ± 13.5
Ko_2015	Australia	NR	482	156	42.5–49.1	326	Age-matched
Niewiadomski_2016	Australia	2007/2008, 2010–2013	155	51	40.0 (11–76)	104	47.5 (24–70)
Niu_2016	China	2008–2013	3,390	678	46.5 ± 15.7	2,712	46.4 ± 15.6
Aniwan_2018	US	2014–2015	1,191	397	34.0 (1.2–93.0)	794	Age-matched
Axelrad_2018	Sweden	2002–2014	462,957	26,450	43 ± 19	436,507	42 ± 20
Amarapurkar_2018	India	1980–2010	2,311	765	40.1 ± 13.6	1,546	38.7 ± 13.6
Park_2019	Korea	2009–2012	23,183,570	9,882	≥18 years	23,173,688	≥18 years
Salgado_2020	Brazil	2015–2017	908	492	Proportion > 40: 67.7%	416	NR
van_der_Sloot_2020	Netherlands	NR	1,669	321	33.6 ± 12.9	1,348	Age-matched
Nguyen_2020	Sweden	2007–2016	133,778	15,951	31.0 (IQR 19–51)	117,827	Age-matched
Blackwell_2021	UK	1998–2016	21,658	10,829	Proportion > 39: 72%	10,829	NR
Oh_2023	Korea	2004–2018	296,304	49,384	45.2 ± 16.8	246,920	Age-matched
Tanaka_2023	Japan	2015–2017	1,049	384	43.0 (29.0–57.5)	665	44.0 (30.0–59.0)
Kondo_2023	Japan	2008–2014	299	132	41.4 (8.7–74.8)	167	44.0 (30.0–59.0)
Sun_2023	UK	2006–2010	430,384	1,576	65.0 (43.0–82.0)	428,808	66.0 (43.0–82.0)
Alperen_2024	Turkey	2020–2021	745	277	36.0 (18.0–75.0)	468	31.0 (18.0–80.0)
Chu_2024	China	2018–2019	328	164	39.0 (33.0–49.0)	164	40.0 (32.7–50.0)
Ho_2025	UK	2006–2010	105,715	373	Average 56	105,342	Average 56
Ahn_2025	Korea	2002–2019	5,524,279	26,332	≥20	5,497,947	≥20

### 3.2 Quality assessment of included studies

Study quality was assessed using the Newcastle–Ottawa Scale (NOS). Among the 30 included studies, NOS scores ranged from 6 to 9, with a median of 8. Overall, 27 studies were classified as high quality, 3 as moderate quality, and none as low quality. These findings indicate that the included studies were generally of good methodological quality and provided a sound evidence base for the present meta-analysis (Table 1 and Appendix 2 of Supplementary material).

### 3.3 Stratified analysis of smoking

Smoking was analyzed according to smoking status. Compared with never smokers, ever smokers had a higher risk of UC, and the direction of association was consistent across case–control and cohort studies (Supplementary Figure S1-A; aOR = 1.53, 95% CI 1.19–1.96; HR = 1.63, 95% CI 1.49–1.79). Former smokers also showed an increased risk of UC (Supplementary Figure S1-C; aOR = 1.49, 95% CI 1.38–1.62; HR = 1.72, 95% CI 1.53–1.94). By contrast, current smoking was associated with a lower risk of UC in case–control studies, whereas the association was not statistically significant in cohort studies (Supplementary Figure S1-B; aOR = 0.67, 95% CI 0.51–0.87; HR = 0.70, 95% CI 0.41–1.20). No significant association was observed for current smoking versus non-current smoking, and this comparison showed substantial heterogeneity (Supplementary Figure S1-D; aOR = 0.37, 95% CI 0.09–1.51).

Leave-one-out sensitivity analysis was conducted only for the case–control subgroup comparing current smokers with never smokers. As shown in Appendix 6-Supplementary Table S1, omission of individual studies one at a time produced pooled estimates ranging from 0.61 to 0.72, with all 95% CIs remaining below 1 and the direction of association unchanged, supporting the overall robustness of this finding. Notably, exclusion of Blackwell_2021 reduced heterogeneity from *I*^2^ = 81.16% to *I*^2^ = 57.04%, suggesting that this study may have been an important source of heterogeneity within this subgroup. The cohort subgroup comparing current smokers with never smokers and the comparison of current smoking versus non-current smoking each included only two studies. Leave-one-out sensitivity analysis was therefore not performed because exclusion of either study would preclude pooled estimation.

### 3.4 Breastfeeding

The association between breastfeeding and UC risk differed by effect estimate type. Meta-analysis of adjusted estimates indicated that breastfeeding was associated with a lower risk of UC (Supplementary Figure S2-A; pooled aOR = 0.75, 95% CI 0.58–0.96, *p* = 0.03; *I*^2^ = 0.0%), whereas meta-analysis of unadjusted estimates showed no significant association (Supplementary Figure S2-B; pooled OR = 1.04, 95% CI 0.77–1.39, *p* = 0.82; *I*^2^ = 0.0%). The discrepancy between the adjusted and unadjusted analyses suggests that confounding control may materially influence the estimated association. Given that factors such as age, socioeconomic status, family environment, and early-life exposures may affect effect estimates in observational studies, adjusted analyses suggested that breastfeeding may be associated with a lower risk of UC. However, because the adjusted and unadjusted analyses yielded inconsistent results and only two studies were included in each subgroup, the available evidence remains limited, and this association requires confirmation in further high-quality studies.

### 3.5 Body mass index

No significant association was found between body mass index (BMI) and the risk of UC (Supplementary Figure S2-C; pooled aOR = 0.59, 95% CI 0.13–2.60, *p* = 0.49). However, heterogeneity was substantial across studies (*I*^2^ = 88.34%, *p* for heterogeneity < 0.001). This heterogeneity may be related to differences in the timing and categorization of BMI assessment across the included case–control studies. In addition, BMI measured close to the diagnosis of UC may be influenced by pre-diagnostic symptoms or disease-related weight changes. Because only two studies were included, leave-one-out sensitivity analysis was not performed, as exclusion of either study would preclude pooled estimation. Taken together, the available evidence does not support a definitive association between BMI and UC risk.

### 3.6 Enteric infection

No significant association was identified between enteric infection and the risk of UC (Supplementary Figure S2-D; pooled aOR = 1.04, 95% CI 0.60–1.80, *p* = 0.90). Heterogeneity was considerable (*I*^2^ = 94.89%, *p* for heterogeneity < 0.001), suggesting limited robustness of the pooled estimate and the need for cautious interpretation. Leave-one-out sensitivity analysis (Appendix 6-Supplementary Table S2) showed that omission of individual studies did not materially change the pooled estimate, which remained statistically non-significant in all analyses, supporting the overall stability of the finding. Nevertheless, the persistently high *I*^2^ indicates that the heterogeneity was not attributable to any single study and may reflect differences across studies in the definition and ascertainment of a history of gastrointestinal infection, as well as the temporal relationship between infection and UC diagnosis. The corresponding exploratory funnel plot is presented in Appendix 7.

### 3.7 Oral contraceptive use

The association between oral contraceptive use and UC risk was examined separately by effect measure type. Meta-analysis of adjusted estimates indicated a borderline inverse association (Supplementary Figure S3-A; pooled aOR = 0.64, 95% CI 0.42–1.00, *p* = 0.05), although heterogeneity was substantial (*I*^2^ = 64.76%, *p* for heterogeneity = 0.01). By contrast, meta-analysis of unadjusted estimates showed no significant association (Supplementary Figure S3-B; pooled OR = 1.22, 95% CI 0.79–1.89, *p* = 0.38), with no evidence of substantial heterogeneity (*I*^2^ = 0.0%, *p* for heterogeneity = 0.39). Taken together, the available evidence does not support a definitive association between oral contraceptive use and the risk of UC. Leave-one-out analysis further showed that omission of any single study did not change the direction of the pooled effect estimate (Appendix 6-Supplementary Table S3), supporting the overall stability of the findings.

### 3.8 Antibiotic exposure

Antibiotic exposure was analyzed separately according to exposure definition. Compared with never/no antibiotic use, any antibiotic use was not significantly associated with UC risk (Supplementary Figure S3-C; pooled aOR = 1.17, 95% CI 0.38–3.60, *p* = 0.78), although considerable between-study heterogeneity was observed (*I*^2^ = 98.14%). Leave-one-out sensitivity analysis for this comparison is presented in Appendix 6, Supplementary Table S4. Compared with never/no antibiotic use, the highest reported category of antibiotic exposure in each study was associated with an increased risk of UC (Supplementary Figure S3-D; pooled aOR = 1.82, 95% CI 1.40–2.37, *p* < 0.001), although substantial heterogeneity was also observed (*I*^2^ = 97.65%). Because the exposure windows and category thresholds used to define the highest exposure category differed across studies, this pooled effect reflects the association between the highest antibiotic exposure category and never/no use within individual studies and does not establish a dose–response relationship based on a uniform exposure threshold. Leave-one-out sensitivity analysis showed that exclusion of Nguyen_2020 reduced heterogeneity to *I*^2^ = 0%, while the direction and statistical significance of the pooled effect remained unchanged (Appendix 6, Supplementary Table S5).

### 3.9 Family history of IBD

Six studies were included in the meta-analysis of family history of IBD and risk of UC. The pooled analysis showed that a family history of IBD was significantly associated with an increased risk of UC (Supplementary Figure S4-A; pooled aOR, 2.88; 95% CI, 1.95–4.25; *p* < 0.001). No substantial between-study heterogeneity was observed (*I*^2^ = 0.0%; Cochran’s Q test, *p* = 0.78), indicating good consistency across the included studies.

### 3.10 Tonsillectomy

Tonsillectomy was not significantly associated with risk of UC (Supplementary Figure S4-B; pooled aOR, 1.52; 95% CI, 0.92–2.50; *p* = 0.10), with substantial heterogeneity across studies (*I*^2^ = 80.96%; Cochran’s *Q* test, *p* < 0.001). Leave-one-out analysis showed that exclusion of Gearry_2010 changed the pooled result to statistical significance and eliminated heterogeneity (*I*^2^ = 0%), indicating that this study was the principal contributor to the observed heterogeneity. Differences in the ascertainment and timing of tonsillectomy relative to UC diagnosis, as well as participant characteristics across studies, may have contributed to this variability.

### 3.11 Appendectomy

The association between appendectomy and risk of UC was examined by effect estimate type. In the adjusted analysis, appendectomy was significantly associated with a reduced risk of UC (Supplementary Figure S4-C; pooled aOR, 0.39; 95% CI, 0.30–0.51; *p* < 0.001), with no evidence of between-study heterogeneity (*I*^2^ = 0.0%; Cochran’s *Q* test, *p* = 0.12). By contrast, the unadjusted analysis showed no significant association (Supplementary Figure S4-D; pooled OR, 0.72; 95% CI, 0.21–2.51; *p* = 0.61) and substantial heterogeneity (*I*^2^ = 95.24%; Cochran’s *Q* test, *p* < 0.001). Overall, the adjusted estimates were more robust, supporting a potential inverse association between appendectomy and UC risk. Leave-one-out analysis (Appendix 6-Supplementary Table S7) showed that the pooled unadjusted result remained unchanged in direction and significance after exclusion of individual studies, with heterogeneity remaining high in all iterations. The substantial heterogeneity in the unadjusted analysis may reflect differences in the timing and indications for appendectomy, as well as variation in age, infection history, and healthcare-related behaviors across studies.

### 3.12 Educational attainment

Using low educational attainment as the reference, high educational attainment was associated with a nonsignificant reduction in UC risk (Supplementary Figure S5-A; pooled aOR, 0.71; 95% CI, 0.49–1.01; *p* = 0.06), whereas intermediate educational attainment was significantly associated with a lower risk of UC (Supplementary Figure S5-A; pooled aOR, 0.43; 95% CI, 0.30–0.62; *p* < 0.001). No between-study heterogeneity was observed in either comparison (both *I*^2^ = 0.0%).

### 3.13 Place of residence

No significant association was observed between place of residence and risk of UC. In the unadjusted analysis, urban/town residence was not associated with UC risk compared with rural residence (Supplementary Figure S5-D; pooled OR, 0.89; 95% CI, 0.71–1.13; *p* = 0.34), with no evidence of between-study heterogeneity (*I*^2^ = 0.0%). Similarly, the adjusted analysis showed no significant association (Supplementary Figure S5-B; pooled aOR, 1.61; 95% CI, 0.94–2.75; *p* = 0.08), although heterogeneity was substantial (*I*^2^ = 83.98%). Leave-one-out analysis (Appendix 6-Supplementary Table S8) showed that exclusion of Bernstein_2006 changed the pooled result to statistical significance (pooled aOR = 1.93, 95% CI 1.10–3.38, *p* = 0.022). Exclusion of LopezSerrano_2010 reduced heterogeneity to *I*^2^ = 54.40%, whereas exclusion of the other studies did not materially alter the overall conclusion. Differences in the classification and timing of residential exposure, as well as adjustment for socioeconomic and lifestyle-related factors, may have contributed to the observed heterogeneity.

### 3.14 Pet exposure

Stratified analysis showed that pet exposure was associated with a reduced risk of UC. Childhood pet exposure was significantly associated with lower UC risk (Supplementary Figure S5-C; pooled aOR, 0.40; 95% CI, 0.22–0.72; *p* = 0.002), with moderate heterogeneity (*I*^2^ = 67.09%). Leave-one-out sensitivity analysis (Appendix 6, Supplementary Table S9) showed that exclusion of Alperen_2024 reduced heterogeneity to *I*^2^ = 0%. Further examination of the original reports suggested that this heterogeneity may be related to differences between Alperen_2024 and the other studies in source populations, control recruitment frameworks, and environmental exposure assessment approaches. Similarly, pet exposure between 5 and 15 years of age was significantly associated with reduced UC risk (Supplementary Figure S5-C; pooled aOR, 0.46; 95% CI, 0.36–0.60; *p* < 0.001), with no evidence of heterogeneity (*I*^2^ = 0.0%). Pooled across all studies, pet exposure remained significantly associated with lower UC risk (Supplementary Figure S5-C; pooled aOR, 0.41; 95% CI, 0.31–0.54; *p* < 0.001; *I*^2^ = 43.60%), with no significant between-subgroup difference (Qb = 0.19, *p* = 0.66).

### 3.15 Egg intake

Two studies were included in the meta-analysis of egg intake and risk of UC. Egg intake was significantly associated with an increased risk of UC (Supplementary Figure S5-E; pooled aOR, 2.84; 95% CI, 1.24–6.53; *p* = 0.01), with moderate heterogeneity across studies (*I*^2^ = 56.54%; Cochran’s *Q* test, *p* = 0.13).

### 3.16 Coffee intake

In the adjusted analysis, coffee intake was significantly associated with a reduced risk of UC (Supplementary Figure S6-A; pooled aOR = 0.23, 95% CI 0.08–0.71, p = 0.01), although heterogeneity was substantial (*I*^2^ = 86.51%; Cochran’s *Q* test, p = 0.01). The included studies were conducted in Japan and Türkiye, and differences in background dietary patterns, coffee consumption behaviors, and participant characteristics may have jointly contributed to the substantial between-study heterogeneity (6, 7). Because only two studies contributed to the adjusted analysis, leave-one-out sensitivity analysis was not performed because exclusion of either study would preclude pooled estimation. By contrast, the unadjusted analysis showed no significant association (Supplementary Figure S6-B; pooled OR = 0.67, 95% CI 0.39–1.16, *p* = 0.15). Given their greater susceptibility to confounding by age, smoking, dietary patterns, and other factors, the unadjusted estimates should be interpreted as supplementary.

### 3.17 Fruit intake

Two studies were included in the meta-analysis of fruit intake and risk of UC. Fruit intake was significantly associated with a reduced risk of UC (Supplementary Figure S6-C; pooled OR, 0.61; 95% CI, 0.45–0.84; *p* = 0.002), with no evidence of between-study heterogeneity (*I*^2^ = 0.0%; Cochran’s *Q* test, *p* = 0.77).

### 3.18 Tea intake

Two studies were included in the meta-analysis of tea intake and risk of UC. Tea intake was not significantly associated with UC risk (Supplementary Figure S6-D; pooled aOR, 0.91; 95% CI, 0.54–1.53; *p* = 0.72), with low to moderate heterogeneity across studies (*I*^2^ = 48.19%; Cochran’s *Q* test, *p* = 0.16).

### 3.19 Alcohol consumption

Stratified analysis showed that light alcohol consumption was significantly associated with a reduced risk of UC (Supplementary Figure S6-E; pooled aOR, 0.47; 95% CI, 0.26–0.88; *p* = 0.02), with substantial heterogeneity (*I*^2^ = 72.83%; Cochran’s *Q* test, *p* = 0.05). By contrast, heavy/daily alcohol consumption or alcohol dependence was not significantly associated with UC risk (Supplementary Figure S6-F; pooled aOR, 0.20; 95% CI, 0.02–1.82; *p* = 0.15), with moderate heterogeneity (*I*^2^ = 61.39%; Cochran’s *Q* test, *p* = 0.11). Leave-one-out sensitivity analysis (Appendix 6, Supplementary Table S10) showed that exclusion of Chu_2024 reduced heterogeneity to *I*^2^ = 0%, identifying this study as the principal contributor to the observed heterogeneity. In contrast, exclusion of van_der_Sloot_2020 rendered the pooled association statistically non-significant, indicating that the result was also sensitive to this study. Further examination of the original reports suggested that, despite harmonization of the comparison as light alcohol consumption versus none/rare alcohol drinking, differences in underlying drinking patterns, beverage types, participant characteristics, and confounding adjustment may have contributed to the observed heterogeneity.

### 3.20 GRADE assessment of the certainty of evidence for factors associated with UC

According to the GRADE assessment (Appendix 4), the certainty of evidence for factors associated with UC was predominantly low or very low, with no high-certainty evidence identified. Of the 22 exposure–outcome associations assessed, 4 were rated as moderate certainty, 5 as low certainty, and 13 as very low certainty. Moderate-certainty evidence supported associations for appendectomy, pet exposure during adolescence, family history of IBD, and intermediate educational attainment. Low-certainty evidence supported associations for childhood pet exposure, ever smoking, former smoking, light alcohol consumption, and egg consumption. The remaining associations, including oral contraceptive use, any antibiotic use, tonsillectomy, place of residence, high educational attainment, BMI, current smoking, current smoking versus non-current smoking, tea consumption, heavy/daily alcohol consumption or alcohol dependence, coffee consumption, and history of gastrointestinal infection, were supported by very low-certainty evidence. Overall, the certainty of evidence was downgraded mainly because the included studies were observational in design and, for several factors, because of limited study numbers, substantial heterogeneity, imprecision (Table 3).

**Table 3:** Summary of GRADE certainty of evidence for dietary, lifestyle, and environmental factors associated with ulcerative colitis.

Certainty of evidence	Environmental factors
Moderate	Appendectomy; Pet exposure during adolescence; Family history of IBD; Intermediate educational attainment
Low	Childhood pet exposure; Ever smoking; Former smoking; Light alcohol consumption; Egg consumption
Very low	Oral contraceptive use; Any antibiotic use; Highest reported category of antibiotic exposure; Tonsillectomy; Place of residence; High educational attainment; BMI; Current smoking; Current smoking vs. non-current smoking; Tea consumption; Heavy/daily alcohol consumption or alcohol dependence; Coffee consumption; History of gastrointestinal infection

## 4 Discussion

**Figure 2:**
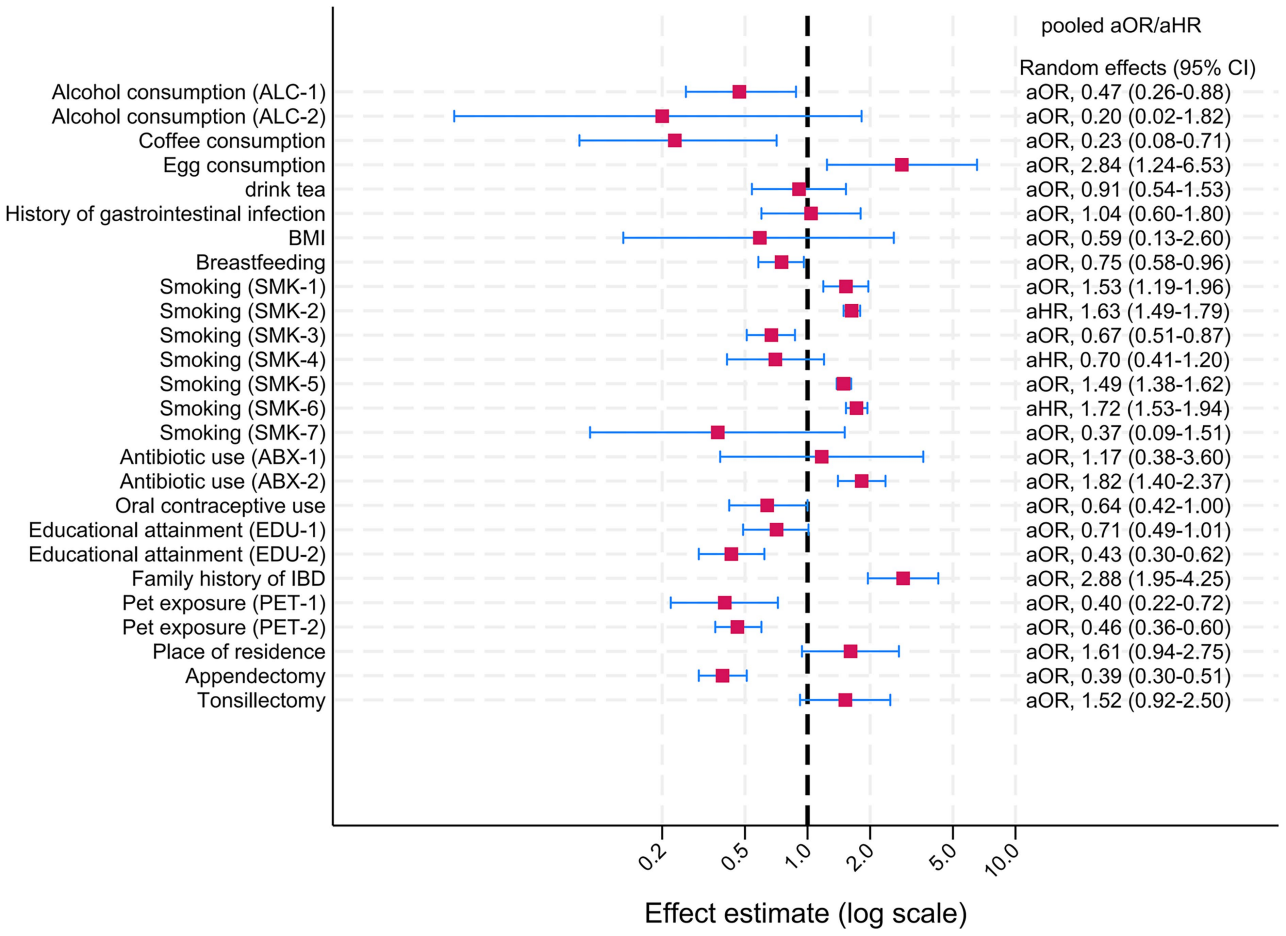
Summary forest plot of pooled effect estimates for dietary, lifestyle, and environmental factors associated with the risk of ulcerative colitis. ALC, 1 any/light drinking, 2 heavy/daily drinking or alcohol dependence; EDU, 1 high, 2 intermediate; PET, 1 childhood exposure, 2 adolescent exposure; SMK, 1 ever, 3 current, 5 former, 7 current vs. non-current; ABX, 1 any use, 2 highest exposure.

By synthesizing observational evidence across dietary, lifestyle, and environmental domains, this study assessed a broad range of factors associated with the risk of UC. As summarized in Figure 2, family history of IBD, the highest reported category of antibiotic exposure, and specific smoking subgroups, particularly ever smoking and former smoking, were associated with an increased risk of UC. Conversely, appendectomy, pet exposure, higher educational attainment, light alcohol consumption, coffee intake, and fruit intake were associated with a lower risk of UC. Current smoking was associated with a lower risk of UC in case–control studies, whereas the association was not statistically significant in cohort studies. Adjusted estimates suggested that breastfeeding might be associated with a lower risk of UC, although the evidence was limited. No clear associations were identified for BMI, oral contraceptive use, tonsillectomy, previous gastrointestinal infection, place of residence, any antibiotic use, tea intake, or heavy alcohol consumption.

Among lifestyle factors, smoking showed the most striking stratified pattern of associations. Former smoking and ever smoking were associated with an increased risk of UC, whereas current smoking showed an apparent inverse association with UC risk. A post-cessation “rebound effect” may partly explain the increased risk observed among former smokers, whereby withdrawal of tobacco exposure may alter immune homeostasis and increase susceptibility to UC in predisposed individuals. Prospective cohort evidence has shown that UC risk increases markedly within 2–5 years after smoking cessation and may persist for a prolonged period thereafter (8). However, the increased risk among former smokers may not be fully explained by short-term biological changes following smoking cessation. Cohort studies have reported associations between UC risk and both smoking intensity and smoking duration among former smokers (9). In addition, former smokers who initiated smoking before adulthood remained at increased risk of UC even after adjustment for smoking amount (10). These findings suggest that the cumulative effects of prior tobacco exposure or persistent biological alterations after smoking cessation may also contribute to this association. On the other hand, the causal interpretation of these findings remains uncertain. Smoking intensity, cumulative exposure, duration since smoking cessation, as well as alcohol consumption, body-weight changes, dietary patterns, and other health-related behaviors, were not consistently controlled for across studies; therefore, residual confounding cannot be excluded. Particularly in retrospective case–control studies, nonspecific gastrointestinal discomfort or changes in health status before diagnosis may influence smoking cessation behavior. Thus, reverse causation may also have contributed to the observed association between former smoking and an increased risk of UC. Mendelian randomization studies have not yet provided sufficient evidence to establish a clear causal relationship between smoking behavior and the development of IBD (11). In contrast, the apparent inverse association observed for current smoking may be related to the ability of nicotine to inhibit NLRP3 inflammasome activation and modulate the gut microbiota (12). Recent studies have also suggested that cigarette smoke may regulate Treg/Th17-related immune responses through GPR15 and exert differential effects in UC and CD models (13). However, these observational findings should not be interpreted as supporting smoking. A more clinically meaningful direction would be to identify the potentially immunomodulatory active components involved and explore safer forms of intervention.

Our findings also suggested a stratified relationship between alcohol consumption and UC risk. Light alcohol consumption was associated with a lower risk of UC, whereas no clear association was observed for heavy alcohol consumption or alcohol dependence, raising the possibility of a dose-dependent effect of alcohol exposure. This issue remains unsettled. Experimental data indicate that alcohol may aggravate DSS-induced colitis and increase susceptibility to infection (14), while other studies suggest that polyphenolic constituents in certain alcoholic beverages may attenuate inflammation through reduction of oxidative stress (15). Taken together, these observations imply that both beverage type and drinking pattern are likely to influence risk. These dimensions should therefore be considered in individualized risk assessment, and further mechanistic work is needed to clarify the dose–response relationship and support more precise prevention strategies for UC.

Dietary factors also showed differential associations with UC risk. Egg intake was associated with an increased risk of UC, whereas coffee and fruit intake were inversely associated with risk; no stable association was observed for tea intake. The potentially protective effect of coffee may be related to chlorogenic acid and other polyphenolic constituents with anti-inflammatory and antioxidant properties (16). Experimental evidence from a DSS-induced Drosophila model of UC-like intestinal injury further supports this possibility. In this model, caffeic acid, a coffee-related phenolic compound, may alleviate UC-like intestinal injury by maintaining mitochondrial homeostasis, limiting apoptosis-related signaling, and suppressing Toll/Imd-mediated immune overactivation (17). The inverse association observed for fruit intake is likewise biologically plausible, given its rich content of dietary fiber, vitamins, and antioxidant compounds, which may contribute to preservation of intestinal barrier function, maintenance of microbial homeostasis, and attenuation of oxidative stress (18). However, the available studies of fruit intake, egg intake, and tea consumption were few and were conducted predominantly in Asian populations, limiting the generalizability of these findings to populations with different dietary patterns and cultural contexts.

The findings for medication use and prior surgical history further underscore the importance of exposure stratification and confounding control in determining the robustness of observed associations. Although no statistically significant association was observed between any antibiotic use and UC risk, the highest reported category of antibiotic exposure in individual studies was associated with an increased risk of UC. Because the exposure windows and category thresholds used to define the highest exposure category varied across studies, it remains difficult to determine whether a uniform dose–response relationship exists between antibiotic exposure and UC risk. This association requires confirmation in further prospective studies using standardized exposure definitions. Previous studies have shown that antibiotics can alter gut microbial composition and diversity and disrupt mucosal immune homeostasis, which may provide biological plausibility for the observed association (19). Among surgery-related exposures, appendectomy showed a relatively consistent inverse association with UC risk, whereas tonsillectomy did not demonstrate a stable association. Previous studies suggest that the appendix contains abundant gut-associated lymphoid tissue and mucosal immune structures involved in antigen sampling and IgA-related immune responses, and that it may also serve as a reservoir for mucus-associated commensal biofilms. Therefore, removal of an inflamed or immunologically active appendix before the onset of UC may reduce abnormal antigenic stimulation and mucosal immune activation, thereby lowering the likelihood of subsequent colonic inflammation (20). Notably, the inverse association for appendectomy was more stable in adjusted analyses, whereas substantial heterogeneity in the unadjusted estimates suggests important confounding by age, surgical indication, infection history, and healthcare-related behaviors. Similarly, oral contraceptive use did not show a stable association with UC risk, and discrepancies between adjusted and unadjusted estimates further indicate susceptibility to confounding by population and behavioral characteristics. Overall, these findings highlight that, in environmental epidemiological studies of UC, both the granularity of exposure definition and the rigor of confounding adjustment directly influence the stability and credibility of the conclusions.

The results for early-life environmental exposures and sociodemographic factors further support the view that susceptibility to UC is not determined solely by adult exposures, but may arise from the long-term accumulation and interaction of multiple factors on a background of genetic predisposition. Early-life environmental exposures, including infant feeding practices and pet contact, may have long-term implications for UC susceptibility through their influence on gut microbial development and immune maturation. Bioactive constituents of breast milk, such as immunoglobulins, lactoferrin, and oligosaccharides, may contribute to early microbial colonization and immune homeostasis, providing biological plausibility for a possible association between breastfeeding and UC risk (21, 22). However, the available epidemiological evidence remains limited and not entirely consistent, and this potential association requires further confirmation.

The broadly consistent direction of association across different childhood windows of pet exposure is likewise compatible with the concept that early environmental microbial contact contributes to immune maturation and the development of immune tolerance. Pet exposure may exert this effect by increasing exposure to a wider diversity of microbial stimuli (23), although the precise mechanisms linking this pathway to UC remain to be established.

Regarding sociodemographic factors, educational attainment was inversely associated with UC risk, whereas place of residence did not show a consistent association. Notably, the positive association between family history of IBD and UC risk was both robust and highly consistent across analyses, reinforcing its status as one of the most reliable epidemiological indicators of UC susceptibility. More broadly, these findings suggest that investigations into the environmental etiology of UC should move beyond a sole focus on adult exposures and more fully incorporate the long-term contributions of early-life and social contextual factors to disease susceptibility.

From a methodological and evidentiary perspective, the GRADE assessment indicates that the certainty of evidence for most exposure–outcome associations was low or very low, with only a minority reaching moderate certainty. This pattern is expected, given that the evidence base consisted primarily of observational studies. Because observational evidence starts at a lower level of certainty and many factors were additionally constrained by small study numbers, substantial heterogeneity, and imprecision, further downgrading of the evidence was unsurprising. These considerations underscore that statistical significance should not be interpreted as equivalent to high-certainty evidence; rather, the current findings are more appropriately viewed as indicators of potential risk signals than as a basis for strong causal inference. The discrepancies observed for several factors between adjusted and unadjusted estimates further highlight the central role of confounding control in studies of dietary, lifestyle, and other external determinants of UC. Age, sex, smoking status, socioeconomic position, dietary patterns, and prior medical history may all influence both exposure and outcome, and inadequate adjustment can materially distort crude estimates. In this context, greater interpretive weight should be given to adjusted effect estimates, which are more likely to reflect the independent association between specific exposures and UC risk.

## 5 Strengths and limitations

A key strength of this study is its focus on incident UC rather than a combined IBD outcome, which helped reduce disease heterogeneity and improve the specificity of the evidence. The review also considered exposures across several relevant domains, allowing the analysis to reflect the multifactorial nature of UC susceptibility rather than being limited to a single exposure category. Where data permitted, adjusted and unadjusted estimates were interpreted separately, and the main findings were examined alongside sensitivity analyses, exploratory assessment of potential small-study effects, and GRADE evaluation. These approaches provided a more structured basis for judging the consistency and certainty of the available evidence.

Several limitations should be acknowledged. Most included studies were observational, so residual confounding and bias could not be fully excluded, and the observed associations should not be interpreted as causal. Differences in exposure definitions, stratification methods, and exposure windows across studies may have affected the comparability of pooled estimates. For some factors, the number of available studies was limited, which reduced the stability of the corresponding results. Although exploratory funnel plots were inspected for selected comparisons, the small number of included studies precluded statistical testing of funnel plot asymmetry. Even so, the combined use of stratified analyses, sensitivity analyses, and evidence certainty assessment helps identify potentially important risk signals and provides direction for future prospective and mechanistic studies.

## 6 Conclusion

UC is a chronic, relapsing inflammatory bowel disease with a complex etiology and substantial impact on quality of life. Identifying factors associated with UC onset is therefore important for risk stratification and early prevention. In this study, family history of IBD, the highest reported category of antibiotic exposure, former smoking, and ever smoking were associated with increased UC risk, whereas appendectomy, pet exposure, higher educational attainment, light alcohol consumption, coffee intake, and fruit intake were associated with reduced risk. Current smoking was associated with a lower risk of UC in case–control studies, whereas the association was not statistically significant in cohort studies. Adjusted estimates suggested that breastfeeding might be associated with a lower risk of UC, although the evidence was limited. No clear associations were observed for BMI, oral contraceptive use, tonsillectomy, previous gastrointestinal infection, place of residence, any antibiotic use, tea intake, or heavy/daily alcohol consumption or alcohol dependence. These findings may help inform early identification of high-risk populations; however, they should be interpreted cautiously in view of the overall certainty of the evidence and require confirmation in future prospective studies before being translated into risk-stratification or prevention strategies.

## Data Availability

The original contributions presented in the study are included in the article/Supplementary material, further inquiries can be directed to the corresponding author.
