# Supplementary material for: Dietary, lifestyle, and environmental factors associated with ulcerative colitis: a systematic review and meta-analysis of observational studies
Source: Front Nutr. 2026 Jul 24;13:1885679. doi: 10.3389/fnut.2026.1885679 (PMC13447159; doi:10.3389/fnut.2026.1885679)
Supplement: Supplementary file 1 [file Data_sheet_1.pdf]

Appendix 1. Search strategies

Table 1: PubMed

No.	Content	Result
#1	"Colitis, Ulcerative"[Mesh]	45494
#2	((((Colitis Gravis[Title/Abstract]) OR (Idiopathic Proctocolitis[Title/Abstract])) OR (Ulcerative Colitis Type[Title/Abstract])) OR (Ulcerative Colitis[Title/Abstract])) OR (Colitis, Ulcerative[Title/Abstract])	59253
#3	("Colitis, Ulcerative"[Mesh]) OR (((((Colitis Gravis[Title/Abstract]) OR (Idiopathic Proctocolitis[Title/Abstract])) OR (Ulcerative Colitis Type[Title/Abstract])) OR (Ulcerative Colitis[Title/Abstract])) OR (Colitis, Ulcerative[Title/Abstract]))	68159
#4	"Risk Factors"[Mesh]	1061407
#5	(((((Populations at Risk[Title/Abstract]) OR (Risk Score[Title/Abstract])) OR (Health Correlates[Title/Abstract])) OR (influencing factor[Title/Abstract])) OR (predisposing factor[Title/Abstract])) OR (environmental exposure[Title/Abstract])	63330
#6	("Risk Factors"[Mesh]) OR ((((((Populations at Risk[Title/Abstract]) OR (Risk Score[Title/Abstract])) OR (Health Correlates[Title/Abstract])) OR (influencing factor[Title/Abstract])) OR (predisposing factor[Title/Abstract])) OR (environmental exposure[Title/Abstract]))	1107455
#7	"Case-Control Studies"[Mesh]	1650338
#8	(((((Case-Control Study[Title/Abstract]) OR (Studies, Case-Control[Title/Abstract])) OR (Case Comparison Studies[Title/Abstract])) OR (cohort study[Title/Abstract])) OR (Closed Cohort Study[Title/Abstract])) OR (Cohort Study, Closed[Title/Abstract])	511276
#9	("Case-Control Studies"[Mesh]) OR ((((((Case-Control Study[Title/Abstract]) OR (Studies, Case-Control[Title/Abstract])) OR (Case Comparison Studies[Title/Abstract])) OR (cohort	1932276

	study[Title/Abstract])) OR (Closed Cohort Study[Title/Abstract])) OR (Cohort Study, Closed[Title/Abstract]))	
#10	((("Colitis, Ulcerative"[Mesh]) OR (((((Colitis Gravis[Title/Abstract]) OR (Idiopathic Proctocolitis[Title/Abstract])) OR (Ulcerative Colitis Type[Title/Abstract])) OR (Ulcerative Colitis[Title/Abstract])) OR (Colitis, Ulcerative[Title/Abstract]))) AND (("Risk Factors"[Mesh]) OR ((((((Populations at Risk[Title/Abstract]) OR (Risk Score[Title/Abstract])) OR (Health Correlates[Title/Abstract])) OR (influencing factor[Title/Abstract])) OR (predisposing factor[Title/Abstract])) OR (environmental exposure[Title/Abstract])))) AND (("Case-Control Studies"[Mesh]) OR ((((((Case-Control Study[Title/Abstract]) OR (Studies, Case-Control[Title/Abstract])) OR (Case Comparison Studies[Title/Abstract])) OR (cohort study[Title/Abstract])) OR (Closed Cohort Study[Title/Abstract])) OR (Cohort Study, Closed[Title/Abstract])))	1502

Table 2: Embase

No.	Content	Result
#1	'ulcerative colitis'/exp OR 'ulcerative colitis'	129,833
#2	'chronic ulcerative colitis':ab,ti OR 'colitis ulcerativa':ab,ti OR 'colitis ulcerosa':ab,ti OR 'colitis ulcerosa chronica':ab,ti OR 'colitis, mucosal':ab,ti OR 'colitis, ulcerative':ab,ti	2,279
#3	#1 OR #2	129,951
#4	'risk factor/exp OR 'risk factor'	1,815,031
#5	'relative risk':ab,ti OR 'risk factors':ab,ti OR 'risk factor':ab,ti	1,431,195
#6	#4 OR #5	2,130,364
#7	'case control study'/exp OR 'case control study'	296,789
#8	'case-control studies':ab,ti OR 'case-control study':ab,ti OR 'control study, case':ab,ti OR 'cohort analysis':ab,ti OR 'analysis, cohort':ab,ti	218,703

#9	#7 OR #8	335,378
#10	#3 AND #6 AND #9	828

Table 3: Cochrane Library

No.	Content	Result
#1	MeSH descriptor: [Colitis, Ulcerative] explode all trees	2389
#2	(Idiopathic Proctocolitis).ti,ab,kw OR (Ulcerative Colitis Type).ti,ab,kw OR (Ulcerative Colitis).ti,ab,kw OR (Colitis Gravis).ti,ab,kw	7382
#3	#1 OR #2	7382
#4	MeSH descriptor: [Risk Factors] explode all trees	38294
#5	(Risk Factor).ti,ab,kw OR (Factor, Risk).ti,ab,kw OR (Risk Factor Scores).ti,ab,kw	5719
#6	#4 OR #5	85083
#7	MeSH descriptor: [Cohort Studies] explode all trees	211449
#8	(Case-Control Study).ti,ab,kw OR (Studies, Case-Control).ti,ab,kw OR (Case Comparison Studies).ti,ab,kw OR (Control Study, Closed).ti,ab,kw	17459
#9	#7 OR #8	223781
#10	#3 AND #6 AND #9	43

Appendix 2. Quality assessment of included studies using the Newcastle–Ottawa Scale

Newcastle–Ottawa Scale (NOS)													
Basic Information				Selection				Comparability	Outcome/Exposure			Summary	
Reference	Study Type	Country	Outcome	S1	S2	S3	S4	Comparability of groups (0-2 分)	OE1	OE2	OE3	Total Score	Quality
GarciaRodriguez_2005	Case-control	UK	UC	1	1	1	1	2	1	1	0	8	High
Bernstein_2006	Case-control	Canada	UC	1	1	1	1	2	1	1	0	8	High
Firouzi_2006	Case-control	Iran	UC	1	0	0	1	2	1	1	0	6	Moderate
deSaussure_2007	Case-control	France	UC	1	1	0	1	2	1	1	0	7	High
Jiang_2007	Case-control	China	UC	1	1	1	1	2	1	1	0	8	High
Sicilia_2008	Case-control	Spain	UC	1	1	1	1	2	1	1	0	8	High
LopezSerrano_2010	Case-control	Spain	UC	1	1	0	1	2	1	1	1	8	High
Gearry_2010	Case-control	New Zealand	UC	1	1	1	1	2	1	1	0	8	High
Hansen_2011	Case-control	Denmark	UC	1	1	0	1	2	1	1	0	7	High
Chan_2013	Cohort	Europe	UC	1	1	1	1	2	1	1	1	9	High
Wang_2013	Case-control	China	UC	1	1	1	1	2	1	1	0	8	High
Ko_2015	Case-control	Australia	UC	1	1	1	1	2	1	1	0	8	High
Niewiadomski_2016	Case-control	Australia	UC	1	1	1	1	0	1	1	0	6	Moderate
Niu_2016	Case-control	China	UC	1	1	1	1	2	1	1	0	8	High
Aniwan_2018	Case-control	United States	UC	1	1	1	1	2	1	1	1	9	High
Axelrad_2018	Case-control	Sweden	UC	1	1	1	1	2	1	1	1	9	High
Amarapurkar_2018	Case-control	India	UC	1	1	1	1	0	1	1	0	6	Moderate
Park_2019	Cohort	Korea	UC	1	1	1	1	2	1	1	1	9	High

Salgado_2020	Case-control	Brazil	UC	1	1	1	1	2	0	1	0	7	High
van_der_Sloot_2020	Case-control	Netherlands	UC	1	1	1	1	2	1	1	0	8	High
Nguyen_2020	Case-control	Sweden	UC	1	1	1	1	2	1	1	1	9	High
Blackwell_2021	Case-control	United Kingdom	UC	1	1	1	1	2	1	1	1	9	High
Oh_2023	Case-control	Korea	UC	1	1	1	1	2	1	1	1	9	High
Tanaka_2023	Case-control	Japan	UC	1	1	0	1	2	1	1	1	8	High
Kondo_2023	Case-control	Japan	UC	1	1	0	1	2	1	1	1	8	High
Sun_2023	Cohort	United Kingdom	UC	1	1	1	1	2	1	1	1	9	High
Alperen_2024	Case-control	Turkey	UC	1	1	0	1	2	1	1	1	8	High
Chu_2024	Case-control	China	UC	1	1	1	1	2	1	1	0	8	High
Ho_2025	Cohort	United Kingdom	UC	1	1	1	1	2	1	1	1	9	High
Ahn_2025	Cohort	Korea	UC	1	1	1	1	2	1	1	1	9	High

Note: For case-control studies: S1 = case definition adequacy, S2 = case representativeness, S3 = control selection, S4 = control definition; OE1 = ascertainment of exposure, OE2 = same ascertainment method, and OE3 = non-response rate. For cohort studies: S1 = exposure group representativeness, S2 = non-exposure group selection, S3 = exposure measurement, S4 = baseline no outcome; OE1 = assessment of outcome, OE2 = duration of follow up, and OE3 = adequacy of follow up. Comparability of groups was scored from 0 to 2 points. Total score ranged from 0 to 9 points. Quality rating was classified as High (7–9), Moderate (4–6), and Low (0–3).

Appendix 3. Supplementary forest plots for dietary, lifestyle, and environmental factors associated with ulcerative colitis

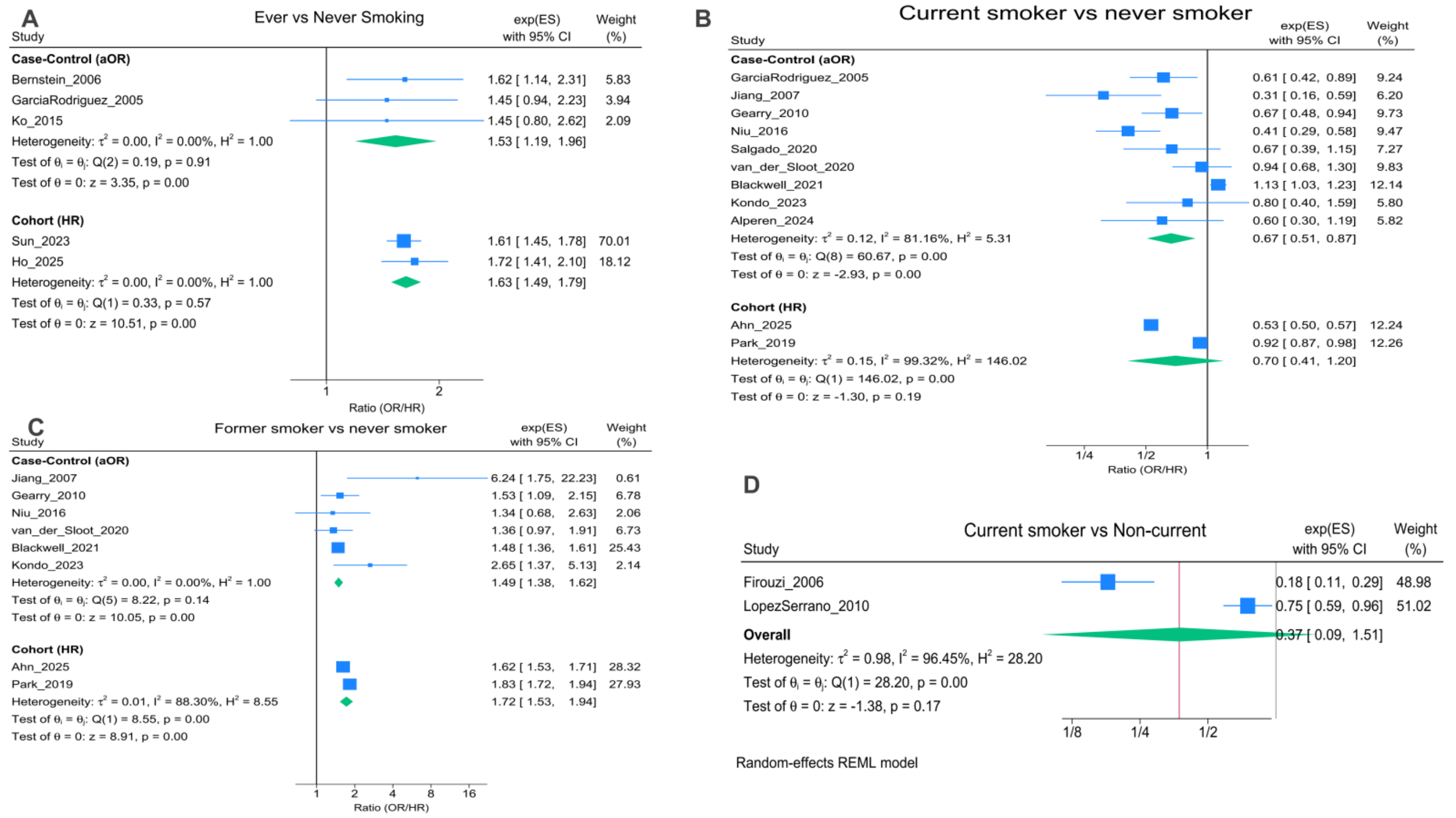

Supplementary Figure S1. Forest plots of the meta-analysis of smoking-related exposures and the risk of ulcerative colitis.

A: Ever smoker vs never smoker; B: Current smoker vs never smoker; C: Former smoker vs never smoker; D: Current smoker vs non-current smoker.

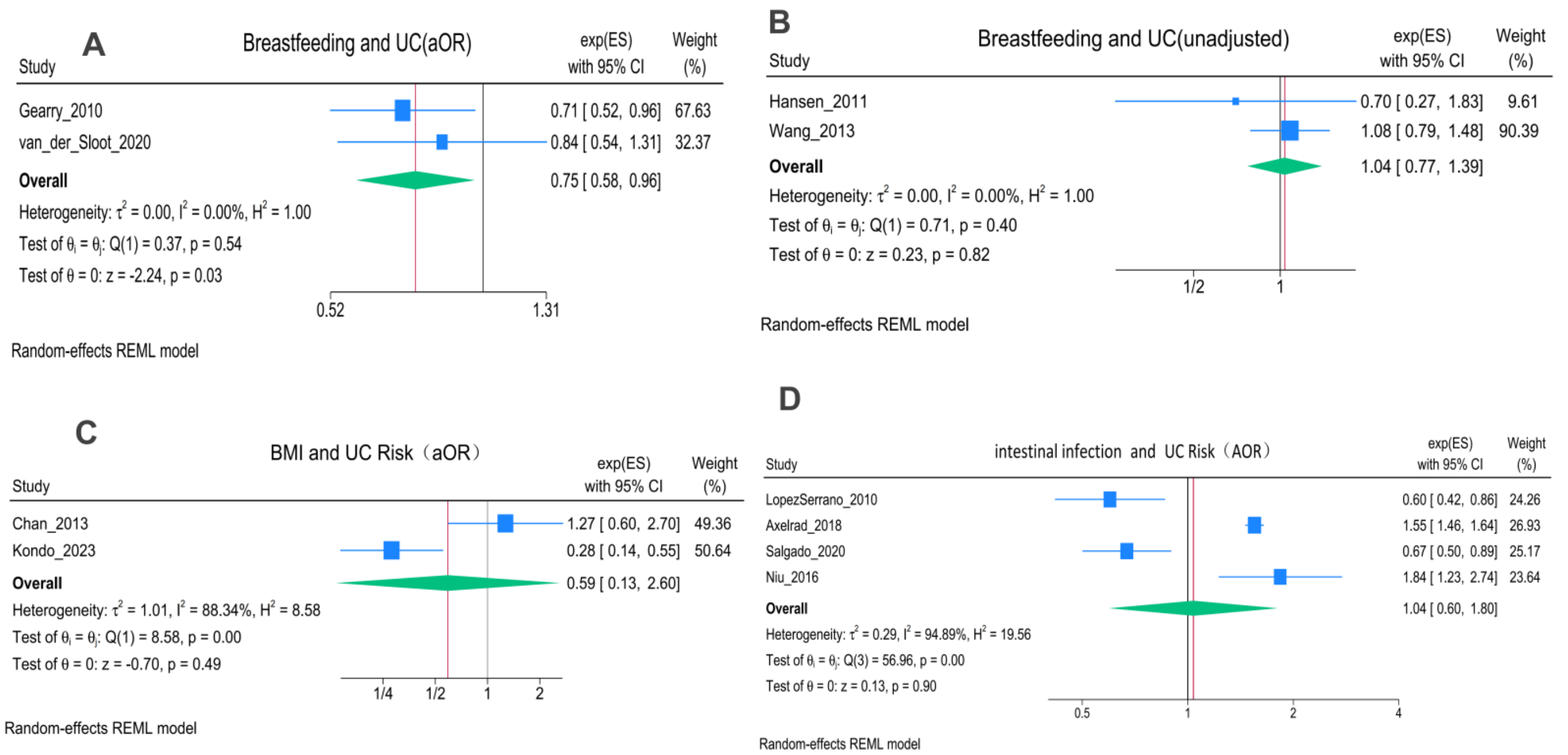

Supplementary Figure S2 .Forest plots of the meta-analysis of breastfeeding, body mass index, intestinal infection, and the risk of ulcerative colitis.

A: Breastfeeding and UC (aOR); B: Breastfeeding and UC (unadjusted); C: BMI and UC risk (aOR); D: Intestinal infection and UC risk (aOR).

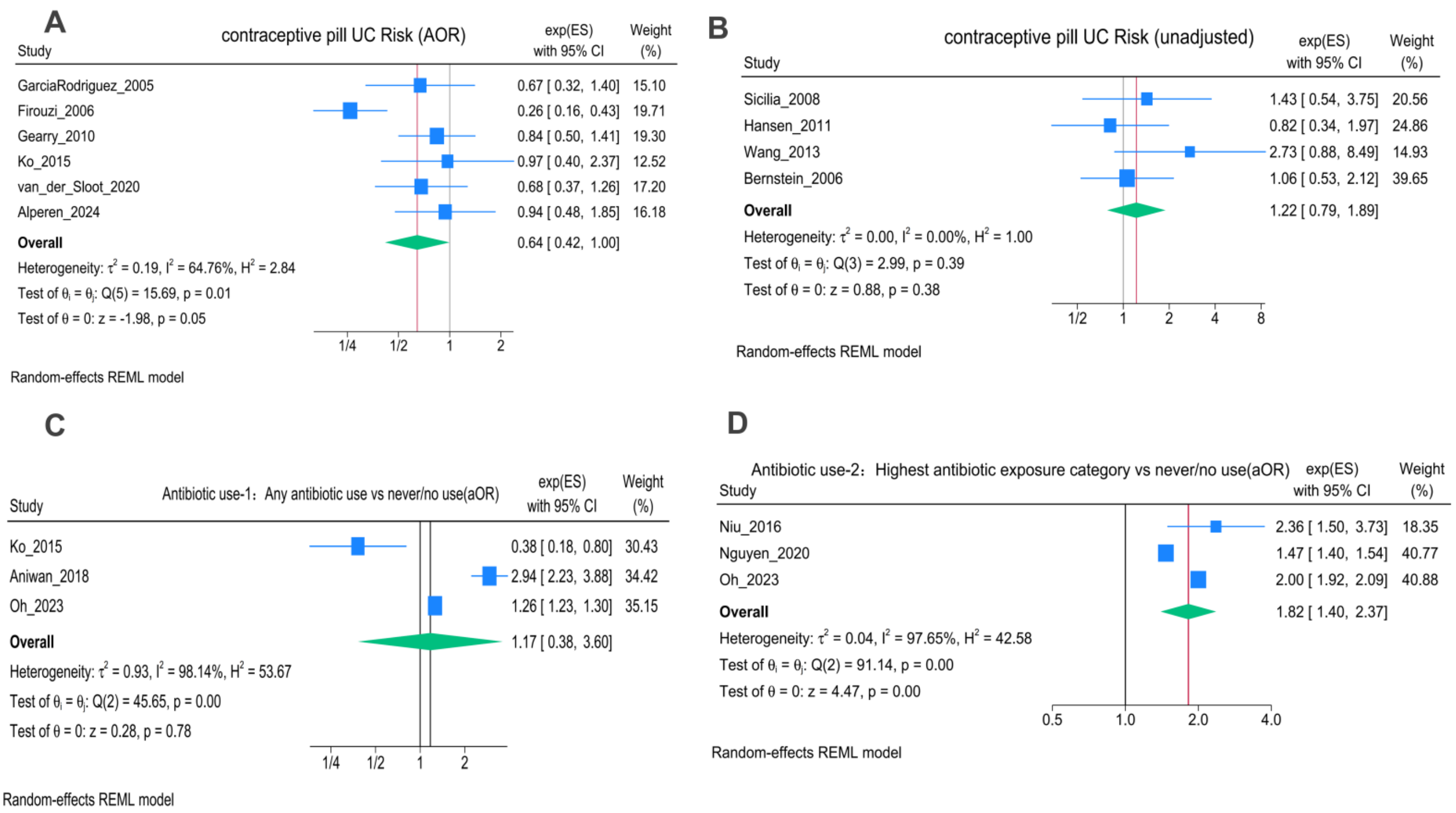

Supplementary Figure S3.Forest plots of the meta-analysis of oral contraceptive use, antibiotic exposure, and the risk of ulcerative colitis.

A: Oral contraceptive use and UC risk (adjusted effect estimates, aOR); B: Oral contraceptive use and UC risk (unadjusted effect estimates, OR); C: Any antibiotic use vs never/no use and UC risk (aOR); D: Highest antibiotic exposure category vs never/no use and UC risk (aOR).

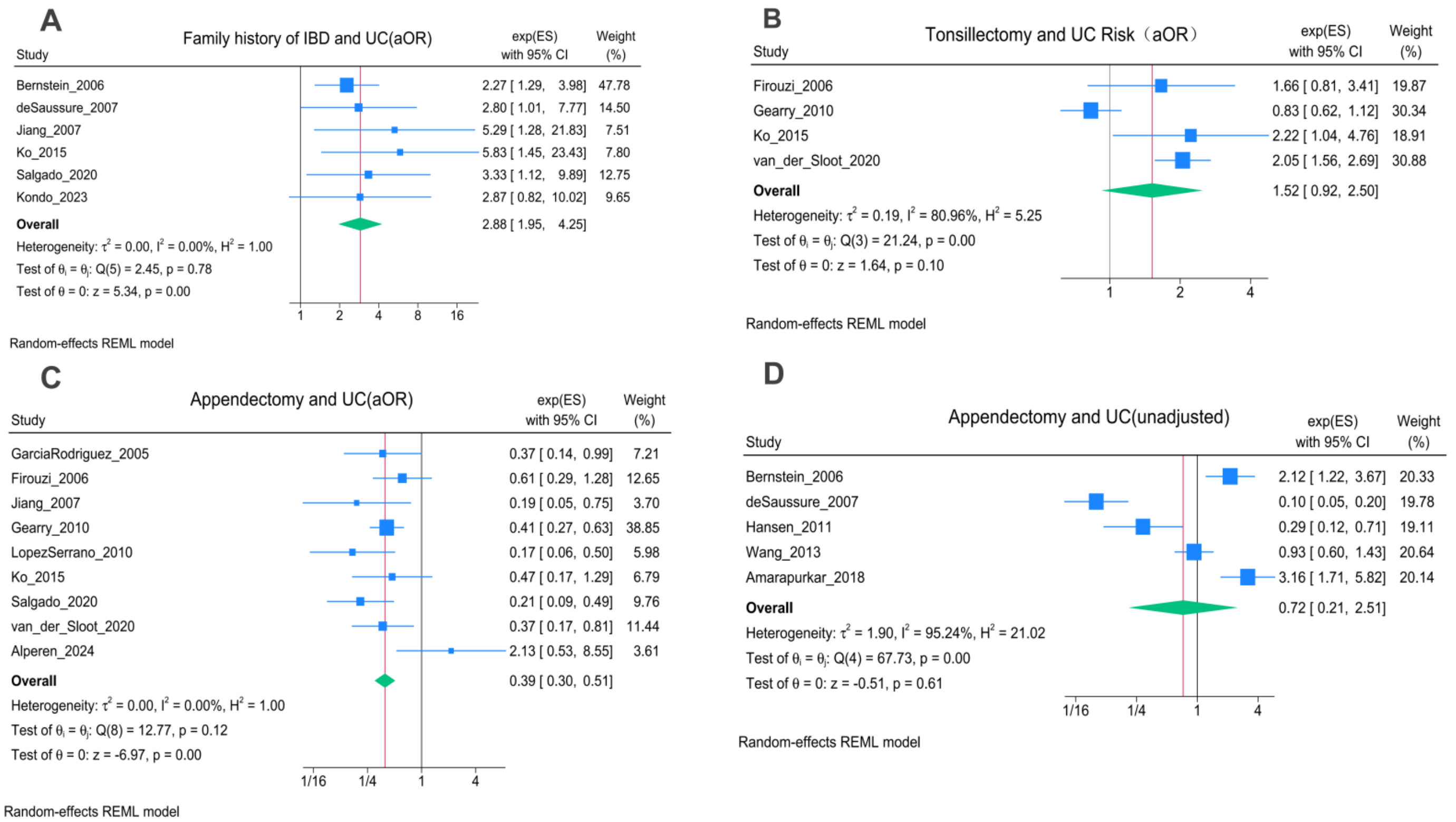

Supplementary Figure S4. Forest plots of the meta-analysis of family history of IBD, tonsillectomy, appendectomy, and the risk of

ulcerative colitis.

A: Family history of IBD and UC risk (aOR); B: Tonsillectomy and UC risk (aOR); C: Appendectomy and UC risk (aOR); D: Appendectomy and UC risk (unadjusted effect estimates, OR).

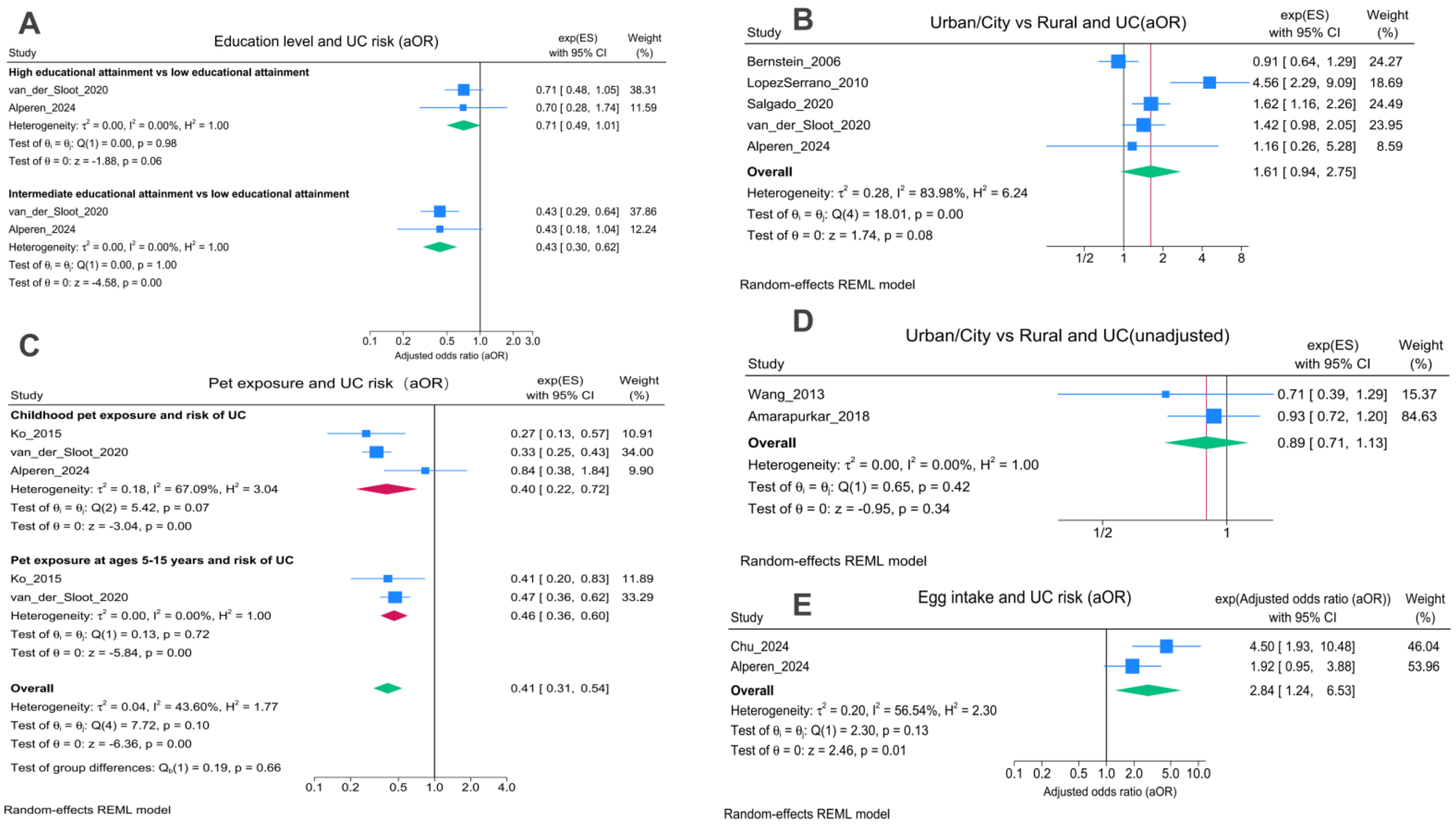

Supplementary Figure S5. Forest plots of the meta-analysis of educational attainment, place of residence, pet exposure, egg intake, and the risk of ulcerative colitis.

A: Educational attainment and UC risk (aOR), including high educational attainment vs low educational attainment and intermediate educational attainment vs low educational attainment; B: Urban/city residence vs rural residence and UC risk (aOR); C: Pet exposure and UC risk (aOR), including childhood pet exposure and pet exposure at age ≤15 years; D: Urban/city residence vs rural residence and UC risk (unadjusted OR); E: Egg intake and UC risk (aOR).

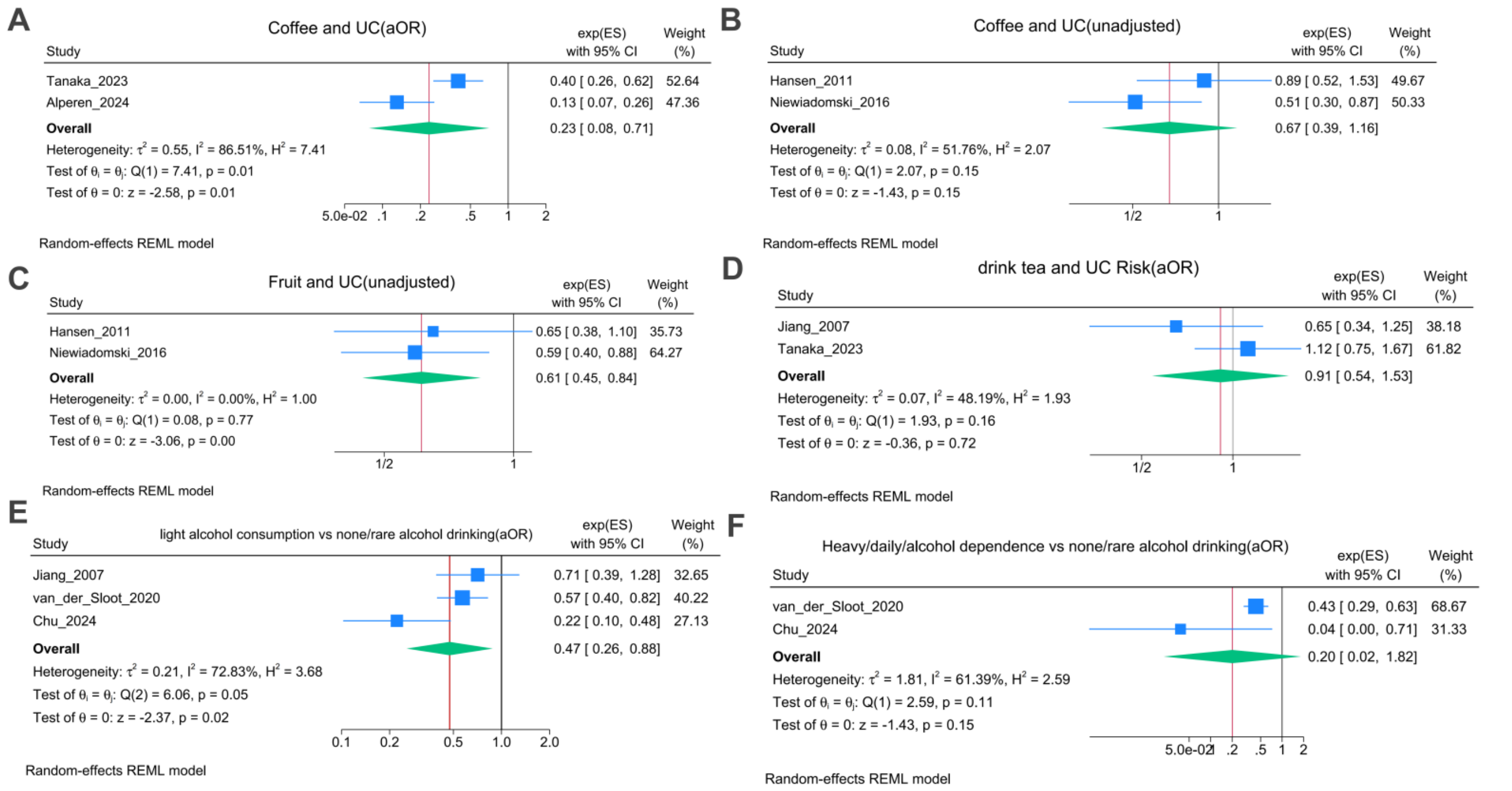

Supplementary Figure S6. Forest plots of the meta-analysis of coffee intake, fruit intake, tea drinking, alcohol consumption, and the risk of ulcerative colitis.

A: Coffee intake and UC risk (aOR); B: Coffee intake and UC risk (unadjusted OR); C: Fruit intake and UC risk (unadjusted OR); D: Tea drinking and UC risk (aOR); E: Light alcohol consumption vs none/rare alcohol drinking and UC risk (aOR); F: Heavy/daily alcohol consumption or alcohol dependence vs none/rare alcohol drinking and UC risk (aOR)

Appendix 4. GRADE assessment of the certainty of evidence for dietary, lifestyle, and environmental factors associated with ulcerative colitis

Exposure	Pooled effect (95% CI)	No. of included studies	Statistical heterogeneity	Quality of evidence (GRADE)
Oral contraceptive use	0.64 (0.42-1.00)	6 studies	$I^2 = 64.76\%$	⨁○○○ (Very low)
Antibiotic use (any vs none)	1.17 (0.38–3.60)	3 studies	$I^2 = 98.14\%$	⨁○○○ (Very low)
Antibiotic use (highest vs none)	1.82 (1.40–2.37)	3 studies	$I^2 = 97.65\%$	⨁○○○ (Very low)
Appendectomy	0.39 (0.30–0.51)	9 studies	$I^2 = 0.00\%$	⨁⨁⨁○ (Moderate)
Tonsillectomy	1.52 (0.92–2.50)	4 studies	$I^2 = 80.96\%$	⨁○○○ (Very low)
Pet exposure (PET-1)	0.40 (0.22–0.72)	3 studies	$I^2 = 67.09\%$	⨁⨁○○ (Low)
Pet exposure (PET-2)	0.46 (0.36–0.60)	2 studies	$I^2 = 0.00\%$	⨁⨁⨁○ (Moderate)
Family history of IBD	2.88 (1.95–4.25)	6 studies	$I^2 = 0.00\%$	⨁⨁⨁○ (Moderate)
Place of residence	1.61 (0.94–2.75)	5 studies	$I^2 = 83.98\%$	⨁○○○ (Very low)
Educational attainment (EDU-1)	0.71 (0.49–1.01)	2 studies	$I^2 = 0.00\%$	⨁○○○ (Very low)
Educational attainment (EDU-2)	0.43 (0.30–0.62)	2 studies	$I^2 = 0.00\%$	⨁⨁⨁○ (Moderate)
BMI	0.59 (0.13–2.60)	2 studies	$I^2 = 88.34\%$	⨁○○○ (Very low)
Smoking (SMK-1)	1.53 (1.19–1.96)	3 studies	$I^2 = 0.00\%$	⨁⨁○○ (Low)
Smoking (SMK-3)	0.67 (0.51–0.87)	9 studies	$I^2 = 81.16\%$	⨁○○○ (Very low)
Smoking (SMK-5)	1.49 (1.38–1.62)	6 studies	$I^2 = 0.00\%$	⨁⨁○○ (Low)
Smoking (SMK-7)	0.37 (0.09–1.51)	2 studies	$I^2 = 96.45\%$	⨁○○○ (Very low)
Tea consumption	0.91 (0.54–1.53)	2 studies	$I^2 = 48.19\%$	⨁○○○ (Very low)
Alcohol consumption (ALC-1)	0.47 (0.26–0.88)	3 studies	$I^2 = 72.83\%$	⨁⨁○○ (Low)
Alcohol consumption (ALC-2)	0.20 (0.02–1.82)	2 studies	$I^2 = 61.39\%$	⨁○○○ (Very low)
Egg consumption	2.84 (1.24–6.53)	2 studies	$I^2 = 56.54\%$	⨁⨁○○ (Low)

Coffee consumption	0.23 (0.08–0.71)	2 studies	$I^2 = 86.51\%$	⨁○○○ (Very low)
History of gastrointestinal infection	1.04 (0.60–1.80)	4 studies	$I^2 = 94.89\%$	⨁○○○ (Very low)

Certainty of evidence was assessed using GRADE. As all included studies were observational, evidence started at low certainty. Certainty was downgraded for risk of bias (based on NOS), inconsistency (I^2 50–75%: −1; ≥75%: −2), and imprecision (95% CI crossing OR=1: −1; crossing with substantial imprecision: −2). Potential small-study effects were explored visually using funnel plots for meta-analyses including at least three studies. Formal statistical assessment of funnel plot asymmetry was not performed because no comparison included 10 or more studies; publication bias was therefore not automatically downgraded. Certainty was upgraded for large effects (pooled OR ≥2.0 or ≤0.5) and for dose–response only when a clear exposure gradient or trend test was reported. Final certainty was rated as high (⊕⊕⊕⊕), moderate (⊕⊕⊕○), low (⊕⊕○○), or very low (⊕○○○).

Appendix 5. Abbreviations for coded exposure comparisons

Code	Comparison
ALC-1	Any/light alcohol consumption vs none/rare alcohol drinking
ALC-2	Heavy/daily alcohol consumption or alcohol dependence vs none/rare alcohol drinking
EDU-1	High vs low educational attainment
EDU-2	Intermediate vs low educational attainment
PET-1	With pets vs without pets in childhood
PET-2	Pet exposure during adolescence (yes vs no)
SMK-1	Ever smoker vs never smoker
SMK-3	Current smoker vs never smoker
SMK-5	Former smoker vs never smoker
SMK-7	Current smoker vs non-current smoker
ABX-1	Any antibiotic use versus never/no use
ABX-2	Highest reported category of antibiotic exposure versus never/no use

Appendix 6. Leave-one-out sensitivity analysis of pooled effect estimates for factors associated with ulcerative colitis

Supplementary Table S1.Current smoker vs never smoker

Study	Pooled OR	95% CI	P-value	I^2 (%)
None (Overall Analysis)	0.67	[0.51, 0.87]	0.003	81.16
Excluding Alperen_2024	0.67	[0.50, 0.90]	0.008	83.94
Excluding Blackwell_2021	0.61	[0.48, 0.78]	< 0.001	57.04
Excluding GarciaRodriguez_2005	0.67	[0.49, 0.91]	0.011	83.04
Excluding Gearry_2010	0.66	[0.48, 0.90]	0.009	82.73
Excluding Jiang_2007	0.72	[0.55, 0.93]	0.012	78.35
Excluding Kondo_2023	0.65	[0.49, 0.88]	0.005	84.00
Excluding Niu_2016	0.72	[0.55, 0.94]	0.016	76.15
Excluding Salgado_2020	0.66	[0.49, 0.90]	0.007	84.04
Excluding van_der_Sloot_2020	0.63	[0.47, 0.85]	0.002	80.62

Supplementary Table S2：Enteric infection

Study	Pooled OR	95% CI	P-value	I^2 (%)
None (Overall Analysis)	1.04	[0.60, 1.80]	0.896	94.89
Excluding LopezSerrano_2010	1.24	[0.68, 2.26]	0.49	94.62
Excluding Axelrad_2018	0.9	[0.45, 1.78]	0.754	91.49
Excluding Salgado_2020	1.2	[0.62, 2.34]	0.59	94.39
Excluding Niu_2016	0.87	[0.48, 1.59]	0.654	95.08

Supplementary Table S3：Oral contraceptive use

Study	Pooled OR	95% CI	P-value	I^2 (%)
None (Overall Analysis)	0.64	[0.42, 1.00]	0.048	64.76
Excluding Alperen_2024	0.6	[0.37, 0.98]	0.042	67.50
Excluding Firouzi_2006	0.8	[0.60, 1.08]	0.14	0.00
Excluding GarciaRodriguez_2005	0.64	[0.38, 1.08]	0.095	71.44
Excluding Gearry_2010	0.61	[0.36, 1.02]	0.06	66.76
Excluding Ko_2015	0.61	[0.38, 0.99]	0.044	69.37
Excluding van_der_Sloot_2020	0.64	[0.38, 1.09]	0.099	70.60

Supplementary Table S4：Antibiotic use-1: any antibiotic use vs never/no use

Study	Pooled OR	95% CI	P-value	I^2(%)
None (Overall Analysis)	1.17	[0.38, 3.60]	0.783	98.14
Excluding Ko_2015	1.9	[0.83, 4.36]	0.129	97.19
Excluding Aniwan_2018	0.74	[0.23, 2.37]	0.606	89.91
Excluding Oh_2023	1.09	[0.15, 8.09]	0.933	96.06

Supplementary Table S5：Leave-one-out sensitivity analysis for the highest reported category of antibiotic exposure versus never/no use and the risk of ulcerative colitis

Study	Pooled OR	95% CI	P-value	I^2 (%)
None (Overall Analysis)	1.82	[1.40, 2.37]	< 0.001	97.65
Excluding Niu_2016	1.72	[1.27, 2.32]	< 0.001	98.88
Excluding Nguyen_2020	2	[1.92, 2.09]	< 0.001	0
Excluding Oh_2023	1.76	[1.12, 2.77]	0.014	75.65

Supplementary Table S6：Tonsillectomy

Study	Pooled OR	95% CI	P-value	I^2 (%)
None (Overall Analysis)	1.52	[0.92, 2.50]	0.1	80.96
Excluding Firouzi_2006	1.5	[0.79, 2.86]	0.22	88.58
Excluding Gearry_2010	2.02	[1.59, 2.57]	< 0.001	0
Excluding Ko_2015	1.39	[0.77, 2.51]	0.275	86.64
Excluding van_der_Sloot_2020	1.34	[0.72, 2.49]	0.363	70.84

Supplementary Table S7：Appendectomy

Study	Pooled OR	95 CI	P-value	I^2 (%)
None (Overall Analysis)	0.72	[0.21, 2.51]	0.611	95.24
Excluding Amarapurkar_2018	0.5	[0.14, 1.86]	0.302	94.56
Excluding Bernstein_2006	0.55	[0.13, 2.37]	0.422	95.32
Excluding Hansen_2011	0.9	[0.20, 3.99]	0.887	96.43
Excluding Wang_2013	0.68	[0.14, 3.38]	0.632	95.63
Excluding deSaussure_2007	1.2	[0.45, 3.21]	0.718	91.05

Supplementary Table S8：Place of Residence

Study	Pooled OR	95% CI	P-value	I^2 (%)
None (Overall Analysis)	1.61	[0.94, 2.75]	0.083	83.98
Excluding Alperen_2024	1.67	[0.91, 3.07]	0.098	89.09
Excluding Bernstein_2006	1.93	[1.10, 3.38]	0.022	76.29
Excluding LopezSerrano_2010	1.28	[0.92, 1.77]	0.143	54.40
Excluding Salgado_2020	1.62	[0.78, 3.40]	0.199	86.17
Excluding van_der_Sloot_2020	1.68	[0.88, 3.23]	0.119	83.34

Supplementary Table S9：Pet Exposure

Study	Pooled OR	95% CI	P-value	I^2 (%)
None (Overall Analysis)	0.4	[0.22, 0.72]	0.002	67.09
Excluding Ko_2015	0.49	[0.20, 1.21]	0.12	79.39
Excluding van_der_Sloot_2020	0.47	[0.16, 1.44]	0.186	76.48
Excluding Alperen_2024	0.32	[0.25, 0.42]	< 0.001	0.00

Supplementary Table S10：light alcohol consumption vs none/rare alcohol drinking

Study	Pooled OR	95% CI	P-value	I^2 (%)
None (Overall Analysis)	0.47	[0.26, 0.88]	0.018	72.83
Excluding Chu_2024	0.61	[0.45, 0.82]	0.001	0.00
Excluding Jiang_2007	0.38	[0.15, 0.95]	0.038	79.08
Excluding van_der_Sloot_2020	0.41	[0.13, 1.27]	0.122	82.01

Appendix 7. Exploratory funnel plots for potential small-study effects in meta-analyses of factors associated with ulcerative colitis

Supplementary Figure S7. Exploratory funnel plots for potential small-study effects in meta-analyses of environmental factors associated with ulcerative colitis.

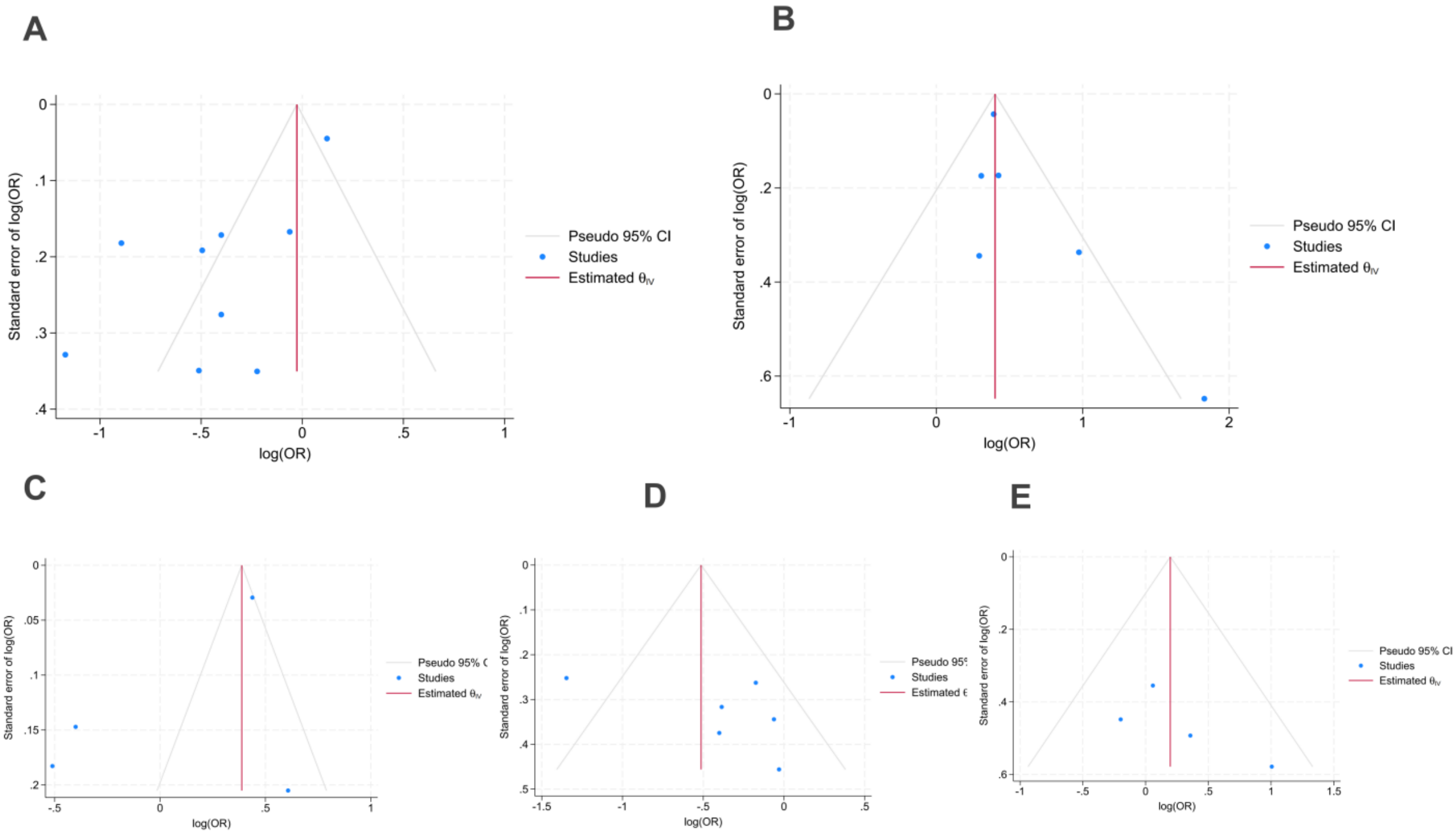

Note: (A) former smoker vs never smoker; (B) current smoker vs never smoker; (C) enteric infection; (D) oral contraceptive use (adjusted effect estimates, aOR); and (E) oral contraceptive use (unadjusted effect estimates, OR).

Supplementary Figure S8. Exploratory funnel plots for potential small-study effects in meta-analyses of factors associated with ulcerative colitis.

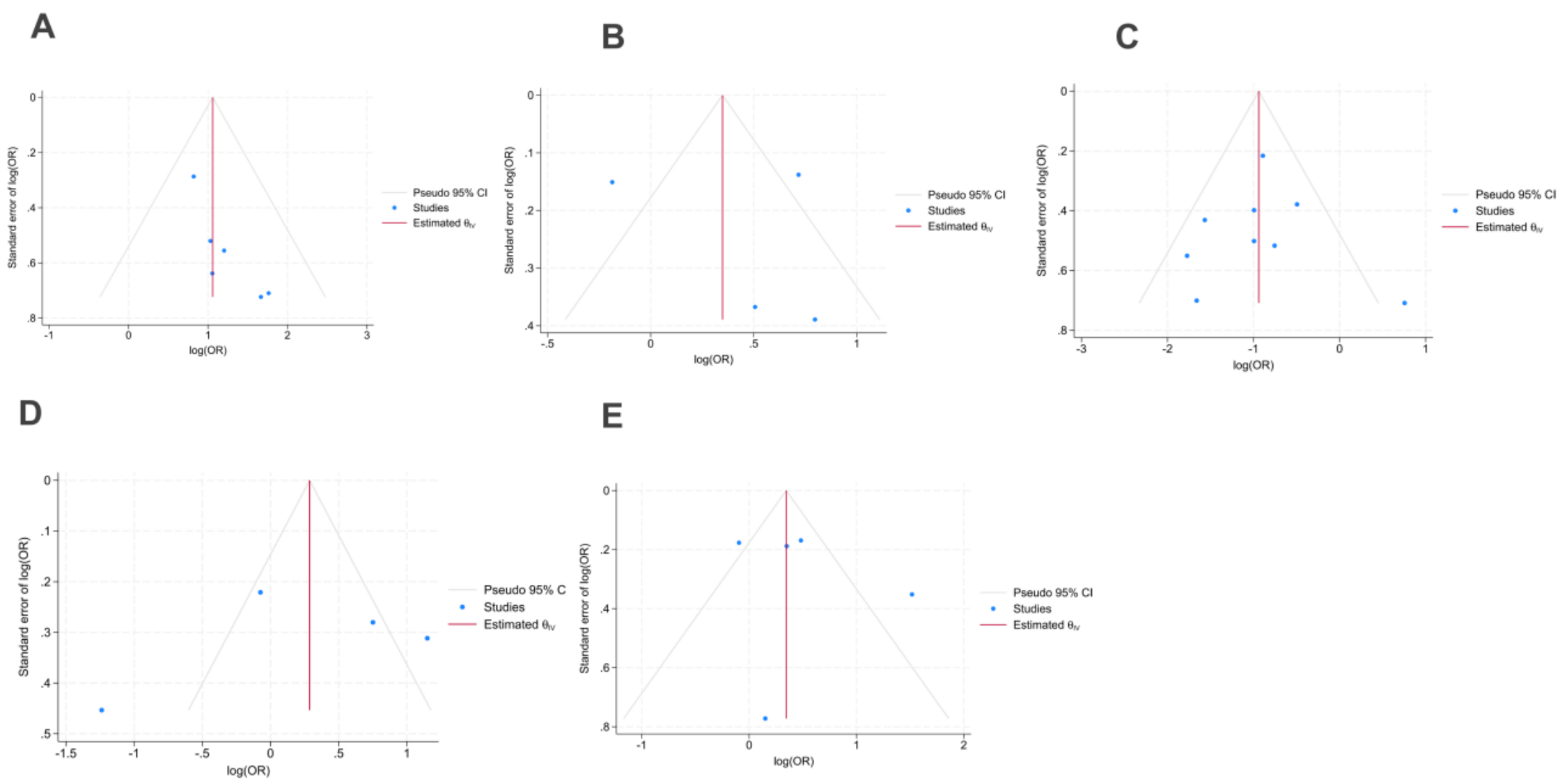

Note: (A) family history of IBD/UC; (B) tonsillectomy; (C) appendectomy (adjusted effect estimates, aOR); (D) appendectomy (unadjusted effect estimates, OR); and (E) place of residence (adjusted effect estimates, aOR).

Supplementary Table 11. Definitions and harmonization of antibiotic exposure comparisons across included studies

Study	Comparison included in the meta-analysis	Exposure definition used in the original study	Reference group	Reason this was not treated as a standardized dose category
Ko_2015	Any antibiotic use vs never/no use	Antibiotic use at ages 0–16 years	No use	Childhood-specific exposure window
Aniwan_2018	Any antibiotic use vs never/no use	History of antibiotic use	Never/no use	Overall exposure history; no common time window
Oh_2023	Any antibiotic use vs never/no use	Any antibiotic use	Never/no use	Broad exposure definition without a standardized frequency threshold
Niu_2016	Highest reported category vs never/no use	Childhood antibiotic use ≥3 times/year	Never use	Frequency-based childhood exposure
Nguyen_2020	Highest reported category vs never/no use	≥3 antibiotic dispensations	None	Dispensation-count metric
Oh_2023	Highest reported category vs never/no use	≥20 antibiotic uses during the reported 2–5-year exposure window	Never/no use	Cumulative-count metric with a different exposure window

Note: For the comparison of the highest reported category of antibiotic exposure versus never/no use, one adjusted effect estimate was extracted from each study by comparing the study-specific highest exposure category with its corresponding never/no-use reference group. Because exposure windows, measurement metrics, and category thresholds differed across studies, the highest exposure categories reported by individual studies were not directly comparable as a common quantitative dose scale.

Supplementary Table 12. Definitions and handling of smoking exposure comparisons across included studies

Smoking comparison	Exposure group	Reference group	Handling in the present meta-analysis
Ever smoking vs never smoking	Participants who had ever smoked, including current or former smokers as defined in the original study	Never smokers	Analyzed as a separate study-reported contrast
Current smoking vs never smoking	Current smokers	Never smokers	Analyzed separately from other smoking contrasts
Former smoking vs never smoking	Former smokers who had quit smoking	Never smokers	Analyzed separately from other smoking contrasts
Current smoking vs non-current smoking	Current smokers	Non-current smokers, which may include never and former smokers	Retained as a distinct contrast and not combined with current smoking vs never smoking

Note: Smoking comparisons were synthesized according to the exposure and reference groups reported in the original studies. Because ever smoking may include current and former smoking, and non-current smoking may include never and former smokers, these categories were not regarded as mutually exclusive across separate meta-analyses. Each study contributed no more than one effect estimate to a given smoking comparison, and estimates from different smoking contrasts were not combined.
